# In silico analysis of Ffp1, an ancestral Porphyromonas spp. fimbrillin, shows differences with Fim and Mfa

**DOI:** 10.1099/acmi.0.000771.v3

**Published:** 2024-07-11

**Authors:** Luis Acuña-Amador, Frederique Barloy-Hubler

**Affiliations:** 1 Laboratorio de Investigación en Bacteriología Anaerobia, Centro de Investigación en Enfermedades Tropicales, Facultad de Microbiología, Universidad de Costa Rica, San José, Costa Rica; 2 Université de Rennes 1, CNRS, UMR 6553 ECOBIO (Écosystèmes, Biodiversité, Évolution), 35042 Rennes, France

**Keywords:** 3D protein modelling, bioinformatics, fimbriae, phylogenomics, *Porphyromonas*

## Abstract

**Background.** Scant information is available regarding fimbrillins within the genus *Porphyromonas*, with the notable exception of those belonging to *Porphyromonas gingivalis*, which have been extensively researched for several years. Besides *fim* and *mfa*, a third *P. gingivalis* adhesin called filament-forming protein 1 (Ffp1) has recently been described and seems to be pivotal for outer membrane vesicle (OMV) production.

**Objective.** We aimed to investigate the distribution and diversity of type V fimbrillin, particularly Ffp1, in the genus *Porphyromonas*.

**Methods.** A bioinformatics phylogenomic analysis was conducted using all accessible *Porphyromonas* genomes to generate a domain search for fimbriae, using hidden Markov model profiles.

**Results.** Ffp1 was identified as the sole fimbrillin present in all analysed genomes. After manual verification (i.e. biocuration) of both structural and functional annotations and 3D modelling, this protein was determined to be a type V fimbrillin, with a closer structural resemblance to a *Bacteroides ovatus* fimbrillin than to FimA or Mfa1 from *P. gingivalis*.

**Conclusion.** It appears that Ffp1 is an ancestral fimbria, transmitted through vertical inheritance and present across all *Porphyromonas* species. Additional investigations are necessary to elucidate the biogenesis of Ffp1 fimbriae and their potential role in OMV production and niche adaptation.

**Impact Statement**

Three distinct fimbriae have been described in *Porphyromonas gingivalis*. Hidden Markov model profiles were used to search genes from these three fimbriae in all the *Porphyromonas* genomes, and it was found that they were differentially present within the genus. Unlike Fim or Mfa, Ffp1 is the only fimbriae common to all *Porphyromonas* monophyletic groups. This gene codes for a stem protein distinct from FimA and Mfa1, and similar to BACOVA_01548. Ffp1 is not present in other bacteria, and seems to be ancestral in *Porphyromonas* spp. As such, studying this gene might help our understanding of niche adaptation and pathogenicity, and other biological process such as outer membrane vesicle production. Characterization of this novel fimbrillin in terms of biogenesis and its involvement in bacterial fitness is lacking and should be addressed.

## Data Summary

External code and software were used as stated in the Methods section, and appropriate literature and/or URLs are provided for all. The authors confirm all supporting data, code and protocols have been provided within the article or through supplementary data files. The hidden Markov model profiles used for this work are publicly available and can be found using DOI: 10.5281/zenodo.10519420[1].

## Introduction

Fimbriae (fibrillae or pili) are adhesins consisting of protein polymers forming filamentous appendages that protrude from the bacterial cell surface. Unlike motility flagella, fimbriae have adhesive properties to attach to surfaces. In Gram-negative bacteria, fimbriae are classified according to their assembly pathways, including the chaperone-usher (CU) pilus system, the type IV pilus and the conjugative type IV secretion pilus [2,3].

In 2016, a new prevalent type V pilus was discovered within the human gut microbiome [4] and was described as a new donor strand-mediated system restricted to the class *Bacteroidia* [3]. This system resembles the CU type, but requires the lipoprotein sorting pathway, and outer membrane proteinases [5].

Type V fimbriae have been mainly studied in *Porphyromonas gingivalis* which classically produces two distinct adhesins, termed FimA (described in 1984 [6]) and Mfa1 (described in 1996 [7]), according to the names of stalk subunits [8]. Both stalk proteins must be processed and matured. They possess long leader peptides [9] that facilitate their transport to the periplasm via the Sec system. Subsequently, they undergo lipid modification and are cleaved by type II signal peptidase [10], followed by a proteolytic maturation achieved by RgpA, RgpB and Kgp proteinases called gingipains [11]. Finally, mature fibrillin monomers polymerize [12]. The genetic loci for both fimbriae are distinct but organized into two clusters: *fimA-E* and *mfa1-5* [13].

In 2017, a third *P. gingivalis* adhesin was described (PGN_1808 in the ATCC 33277 strain or PG1881 in the W83 strain) and termed Ffp1 for filament-forming protein 1 [14]. It corresponds to filaments 200–400 nm in length and 2–3 nm in diameter that can be degraded, unlike FimA or Mfa1, by detergents and temperature into 50 kDa monomers [15]. Ffp1 is among the exclusive repertoire of proteins within the order *Bacteroidales* and was described as conserved across *Porphyromonas* and *Bacteroides* [16,17]. This protein was identified among the outer membrane proteins and especially the O-glycoproteome of *P. gingivali*s [18] and was described as essential in the production of outer membrane vesicles (OMVs), as the Ffp1 null-mutants exhibited a 55 % reduction in OMV production compared to the wild-type strain [14]. Moreover, a recent study indicated a connection between Ffp1 and the production of sphingolipids (SLs). In the absence of SLs, *P. gingivalis* generates OMVs without Ffp1, whereas OMVs containing SLs exhibit an enrichment of Ffp1. Interestingly, these SL-containing OMVs limit host inflammation [19].

The Ffp1 C-terminal region is homologous to type IV fimbriae from *Bacillus* spp. [16], and its sequence bears a significant similarity to the adhesion protein BACOVA_01548 (PDB ID: 4rfj) from *Bacteroides ovatus* [4]. Structural modelling suggests a donor strand-mediated assembly mechanism [15], which would classify Ffp1 as a new type V pilin [14]. However, unlike FimA or Mfa1, no accessory component has yet been identified for Ffp1 despite its apparent co-expression as an operon with three upstream genes, annotated as a Cys-RNAt ligase, a patatin (lipase) and a glycosyl transferase. This co-transcription suggests the involvement of these four proteins in the same biochemical pathway or utilization of the same substrates/transporters, albeit without physical interaction [15].

To date, Ffp1 has been the subject of few studies limited to *P. gingivalis*, only on two reference strains, ATCC 33277 and W83, and no information is available for the other 21 *Porphyromonas* species. At the genus level, knowledge for non-*P. gingivalis* Ffp1 or other fimbriae is scarce, except for description of FimA-like and Mfa1-like fimbriae in *P. gulae*, a closely related species to *P. gingivalis* [20,21], and reports indicating fimbriation in *P. circumdentaria*, *P. macacae* and *P. asaccharolytica* [22,24], without further characterizations.

In this context, the aim of this study is to complete this knowledge gap and to investigate the distribution and diversity of type V fibrillin, particularly Ffp1, in the genus *Porphyromonas*. To do so, we performed an *in silico* analysis of the type V fimbrillin locus in all 144 available genomes of *Porphyromonas*, investigating their presence/absence and then focus on Ffp1 diversity, and 3D predicted structure.

## Methods

### *Porphyromonas* taxogenomics

All 144 *Porphyromonas* genomes (Table S1, available in the online version of this article) were automatically downloaded from the NCBI RefSeq database [25] (release 217, 8 March 2023) using the ncbi-genome-download script v0.2.12 [26]. Unannotated metagenome-assembled genomes (MAGs) with inconsistent taxonomic labels were not considered. To categorize all genomes into reliable groups, genomic data-driven taxonomic confirmation and/or assignment were performed. To confirm the assignment of genomes with a species name, we conducted a comparison of three metrics: (i) the 16S rRNA gene percentage identity (when annotated), evaluated using a threshold of 98.65 % [27]; (ii) the digital DNA–DNA hybridization distance (DDH) using the GGDC v2.1 [28] and ggdc-robot script v0.04 [29], with the default threshold of 70 % using formula 2 [28,30, 31]; and (iii) the whole genome average nucleotide identity (gANI), calculated using FastANI v1.34 [32] with a threshold of 96 % for species demarcation [33]. In case of a disagreement between these three metrics, we combined alignment fraction values (AFs) with gANI using 60 and 96.5 % as threshold values respectively, to assign a genome pair to the same species [34]. Additionally, when needed, we also used OrthoANI v0.93.1 [35] to measure and visualize the overall similarity between some *Porphyromonas* species.

For the genomes without a specified species name (i.e. *Porphyromonas* sp.), as most of them originated from environmental samples (human- or animal-associated habitats) and are often highly fragmented, it was crucial to ensure that they were not contaminated and do not correspond to genome assemblies containing a mixture of different species. This genomic homogeneity was evaluated with Kraken2 v2.1.3 [36] using the non-redundant nucleic database (updated April 22). Only assemblies that consisted of over 80 % of *Porphyromonas* content and/or larger than 80 % of the expected average genome size (2.5 Mb) were retained for our analysis. Their affiliation to the genus *Porphyromonas* was first confirmed using the fIDBAC server[37,38] and their position within the *Porphyromonas* taxonomy was validated using an OrthoFinder v2.5.5 [39] rooted species tree [40]. This tree was reconstructed using all *Porphyromonas* sp. (*P*. sp.) and one reference genome per *Porphyromonas* species (see Table S1) and was visualized using FigTree v1.4.4 [41]. For each branch, one or several *P.* sp. were associated with a *Porphyromonas* species through ANI and DDH, employing the same thresholds as previously described.

### *Porphyromonas* fimbriae identification and classification

1. **Dataset construction:** Individual sequences from type V fimbriae (FimABCDE, Mfa12345 and Ffp1) were manually extracted from the 59 *P*. *gingivalis* genomes and each one was used as query to identify homologous sequences all in the genomes of other *Porphyromonas* spp. using BlastP [42] (identity ≥30 %; query coverage ≥60 %; e-value <10e^−5^). All sequences were grouped as dataset 1.
2. **Functional domain-based screening:** Dataset 1 was subjected to analysis using InterProScan [43] to identify all protein domains associated with those sequences. The resulting domains were searched in the complete orfeomes of *Porphyromonas* downloaded from PATRIC v3.6.6[44,45], using ‘hmmsearch’ from HMMER v3.3.1 [46,47] and the hidden Markov models (HMMs) from the Pfam v33.1 database [48] (May 2020). Sequences harbouring the targeted domains with an e-value <10e^−06^ were retained and grouped into dataset 2.
3. **Protein clustering, biocuration and HMM profile construction:** Dataset 2 was clustered with MMseqs2 v15-6f452 [49] via the ‘easy-cluster’ command. Each cluster obtained underwent manual biocuration after multiple alignment using Clustal Omega[50,51] and any missing genes were annotated. Subsequently, for each cluster, using HMMER, the multiple alignments were converted from FASTA format to Stockholm format with ‘esl-reformat’ command and HMM profiles were generated using the ‘hmmbuild’ command with default settings. Clustering and HMM profile creation was first performed on raw data and then refined on biocurated data.
4. **Final classification:** The obtained HMM profiles (see Supplementary material) were used to identify and classify all fimbrillins within the *Porphyromonas* orfeomes, downloaded from the PATRIC database, using the ‘hmmsearch’ command from the HMMER package.
5. ***In silico*****analysis of*****Porphyromonas*****fimbrillins:** Geneious Prime v2023 [52] was used to visualize the genomic context of each identified fimbrillin. Biocuration for start codons was proposed, based on sequence homology, to optimize the prediction of signal peptidase II (SPII) signal peptide and the cleavage site positions. The N-terminal region was identified using *charge* (window size=3) from EMBOSS v6.6.0 [53], the H hydrophobic region was characterized with a Kyte-Doolittle hydropathy plot made with ProtScale [54,55] (window size=3), and the cleavage site was confirmed by SignalP v6.0 [56] and LipoP v1.0 [57]. Palmitoylation in the lipobox cysteine residue was verified using CSS-Palm v4.0 [58]. Protein sizes were represented using violin plots (geom_violin) and/or boxplots (geom_box), both functions from the ggplot2 package [59].

For each fimbrillin family, a multiple alignment was performed using MAFFT v7.490 (L-INS-I algorithm and BLOSUM62 matrix; gap open penalty and offset value by default) [60]. This alignment was visualized in two dimensions using Alignmentviewer v1.1 [61] which employs the UMAP algorithm [62] and Hamming distance to cluster aligned sequences. Phylogenetic trees were calculated using FastTree v2.1.11 [63], PhyML v3.3 [64] and RaxML v4.0 [65] with default parameters.

The taxonomic distribution of fimbrillin genes was analysed across a phylogenetic tree reconstructed using OrthoFinder based on the pangenomes of all confirmed *Porphyromonas* species groups and visualized using FigTree. The phylogenetic reconstruction was performed both using native and mature proteins (i.e. excluding their signal peptides) using RaxML (evolution model GAMMA LG and 100 bootstraps). Robinson-Foulds, Nye Similarity and Jaccard Robinson Foulds distances between the phylogenetic trees were calculated using the TreeDist [66] R library and tanglegrams were created with the R package phytools [67] (scripts TREE.R and Tanglegram.R).

**6.3D modelling:** Secondary protein structure was predicted with PSIPRED v4.0 [68,69] and Phyre2 v2.0[70,71]. 3D structures of Ffp1 mature proteins were modelled, based on homology modelling, using Robetta [72,73] and the RoseTTAFold method, as well as Phyre2. The quality of all five 3D models generated by Robetta for each Ffp1 protein was assessed and validated using two quality calculation tools: ERRAT [74,75] and Verify3D[76]. The most accurate predicted structure was chosen and superposed to the best model target, found by VAST+[77,78], Phyre2 and iPBA [79,80]. The RMSD value [81] as well as the number and percentage of aligned residues were retrieved and compared to Phyre2 results. RMSD values of <3 Å were considered significant between Ffp1 predicted structure and 3D models [82].

## Results

### *Porphyromonas* taxogenomic assignment

The 144 *Porphyromonas* genomes studied in this work (Table S1) were predominantly in draft form (85 % of the genomes), with only six out of the 17 analysed species possessing at least one complete genome.

The taxogenomic assignment for the genomes classified into the 17 *Porphyromonas* species was verified (Table S1). The *Porphyromonas* species *P. loveana* and *P. pasteri* have only one representative genome and therefore cannot be verified intra-specifically. For the other species, intra-specific analysis combining ANI, 16S rRNA and DDH comparisons (Fig. S1A) showed no anomalies for taxonomic placements, except for *P. uenonis*, *P. somerae* and *P. canoris*.

Firstly, for *P. uenonis*, the differences in metrics reflect a significant distance between strain 60-3 and the two other strains (Fig. S1A and S1B). Strain 60-3 was analysed using Kraken2 and it was concluded that *P. uenonis* 60-3 belongs to the genus *Porphyromonas* but not to *P. uenonis* (Fig. S2). This genome has been retained for the study but as an unclassified *Porphyromonas*, denoted as PSP_60-3 (Table S1).

Secondly, in the case of *P. somerae* KA00683, Kraken2 analysis indicates a genomic mixture, and our taxonomic analysis separates this strain from the other two within the species (Fig. S1B). Consequently, we have opted not to include *P. somerae* KA00683 in our study (Table S1).

Finally, regarding *P. canoris* (two genomes), the difference in the 16S rRNA gene sequences was associated with a longer gene in one strain (Fig. S1C). It is impossible to determine whether this difference represents genuine genomic diversity or a sequencing error; we consider both genomes as belonging to *P. canoris* (Table S1).

**Fig. 1.**
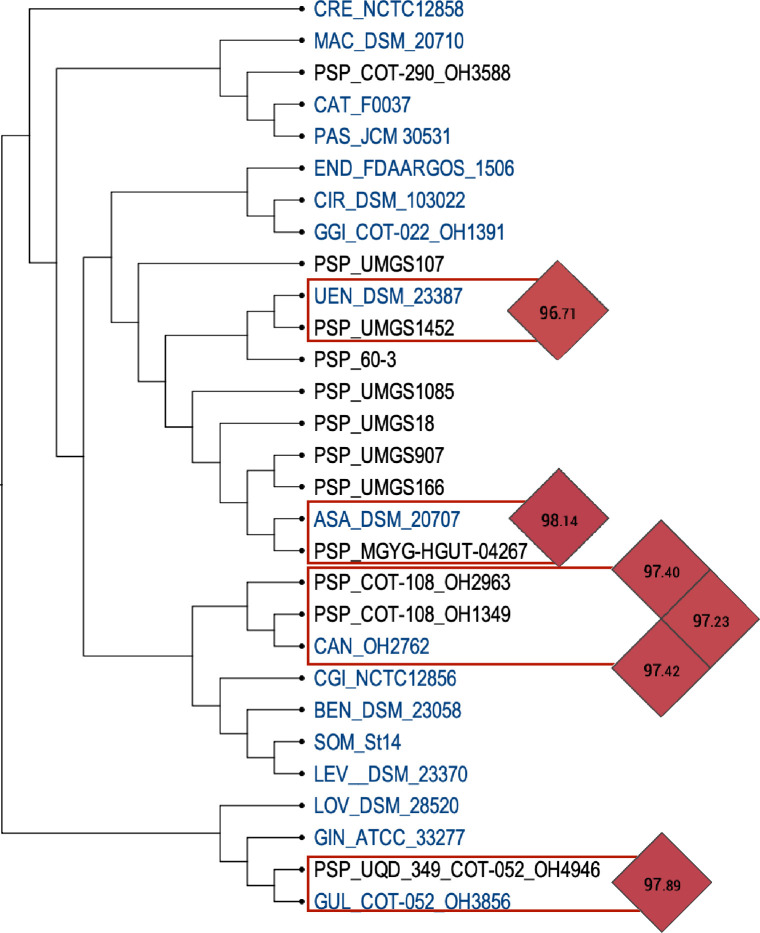
Phylogenetic species tree derived from OrthoFinder analysis. This tree was used to place some *Porphyromonas* spp. within UEN (UMGS1452), ASA (MGYG-HGUT-0467), CAN (OH2963 and OH1349) and GUL (OH4946), after confirmation via OrthoANI. Three-letter code acronyms correspond to ASA: *P. asaccharolytica*; BEN: *P. bennonis*; CAN: *P. canoris*; CAT: *P. catoniae*; CGI: *P. cangingivalis*; CIR: *P. circumdentaria*; CRE: *P. crevioricanis*; END: *P. endodontalis*; GGI: *P. gingivicanis*; GIN: *P. gingivalis*; GUL: *P. gulae*; LEV: *P. levii*; LOV: *P. loveana*; MAC: *P. macacae*; PAS: *P. pasteri*; SOM: *P. somerae*; and UEN: *P. uenonis*.

Furthermore, 28 *Porphyromonas* genomes lacked a species label. All genomes were examined using Kraken2, and genomes with less than 80 % of *Porphyromonas* reads and/or that reconstructed less than 80 % of *Porphyromonas* average genome size (2.5 Mb) were excluded from the study (Fig. S2 and Table S1). Consequently, 17 strains were omitted from this study (Table S1). Among the 11 remaining *Porphyromonas* sp., their placement in the OrthoFinder species tree based on ANI/DDH metrics (Fig. 1) allowed us to assign genomes to: *P. gulae*, *P. asaccharolytica*, *P. uenonis*, and two genomes to *P. canoris* (Fig. 1 and Table S1). Finally, there were six *P*. sp. genomes that could not be assigned to any specific group and were individually examined (unassigned, Table S1).

After completing this taxogenomic biocurated analysis, our study retained a total of 126 *Porphyromonas* genomes clustered into 24 groups (comprising 17 species and seven *P*. sp. singletons), unequally distributed between the genus, ranging from 59 genomes for *P. gingivalis* (almost half of all available genomes in the genus) to just one genome for *P. loveana*, *P. pasteri* and each *Porphyromonas* sp. (PSP).

### FFp1 is the only fimbrillin common to all *Porphyromonas*

**Fig. 2.**
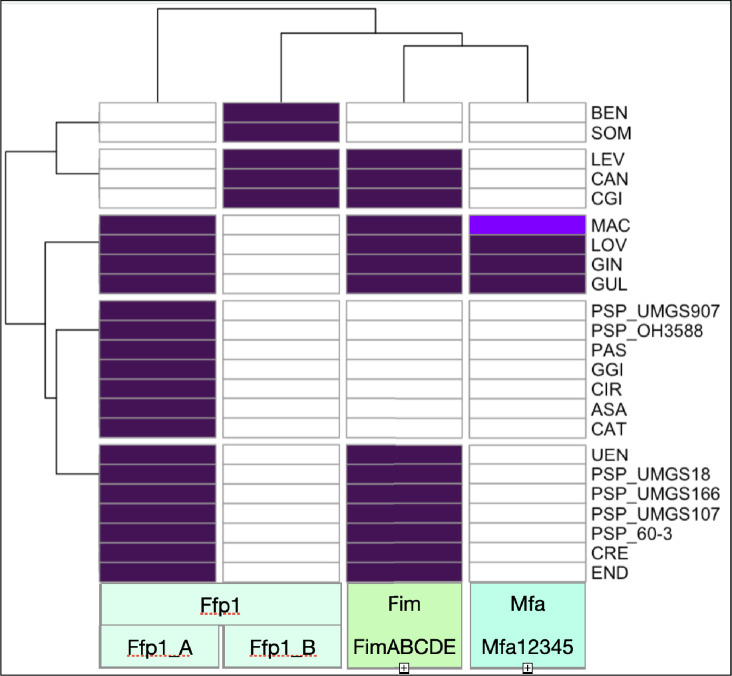
Heatmap depicting the presence/absence of fimbrillins. The heatmap scale colour indicates whether fimbriae systems (FimABCDE, Mfa12345, or Ffp1_A or B) were detected: white (absence), dark purple (presence as one *locus*) and light purple (presence as two *loci*).

Screening and clustering fimbrillin genes from *Porphyromonas* genomes resulted in the definition of 12 HHM profiles, one for each gene in either FimABCDE or Mfa12345, and two for Ffp1. Searching for sequence similarity in each *Porphyromonas* orfeome, using each of the 12 HHM profiles, enabled the identification and classification of these three fimbriae systems in all *Porphyromonas* genomes (Fig. 2).

### *fimABCDE* locus

For the FimABCDE proteins (Fig. S3A), an expected value (E-value) calibration was performed and set to a minimum threshold of e^−100^ for each of the five profiles. Using this threshold, the detection of the locus *fimABCDE* exhibited both sensitivity and specificity, perfectly correlating with presence/absence of each gene.

In each genome, these genes are co-localized and organized into operons, with an average size of 7.3 kb. Of all the genomes, two stand out as outliers: *P. gingivalis* A7436 due to an IS5 family transposase ISPg8 insertion in *fimC*, and *P. uenonis* UMGS1452 for which the locus remains incomplete because it is located at the end of a contig.

It is noteworthy that all *P. macacae* strains possess two complete *fimABCDE* loci, a unique feature in *Porphyromonas*. This duplication raises questions about the redundancy or functional complementarity of both *loci*, especially as *P. macacae* JCM15984 has a pseudogenized *fimE* in locus 1 and a pseudogenized *fimD* in locus 2.

The utilization of HMM profiles in our search strategy allows for the rapid and unambiguous identification and classification of fimbrial genes, even in cases with low mean amino acid percentage identities: 52.3 % (FimA), 63.7 % (FimB), 56.7 % (FimC), 48.2 % (FimD) and 49.8 % (FimE). Additionally, the annotations of FimABCDE proteins are inconsistent, with the majority being labelled as hypothetical proteins or simply categorized as fimbrial proteins without any additional characterization (Fig. S3B). As such, ontology searches are almost impossible.

Moreover, the establishment an E-value threshold facilitates pinpointing abnormalities. For instance, in *P. gingivalis*, for the FimB HMM profile, the E-value is greater than the established threshold due to a nonsense mutation in *fimB* for the ATCC 33277 strain [83], and this gene is annotated as two genes (PGN_0181, e-value=2.8e^−63^ and PGN_0182, e-value=1.4e^−55^). The same case occurs in *P. uenonis*, for the FimE HMM profile, due to the incompleteness of this gene (at the end of contig) for the UMGS1452 strain.

In every analysed *Porphyromonas* genome, the *fimABCDE* locus is consistently present, with only nine groups lacking this operon: *P. asaccharolytica*, *P. bennonis*, *P. catoniae*, *P. circumdentaria*, *P. gingivicanis*, *P. pasteri*, *P. somerae*, *P*. sp. OH3588 and * P*. sp. UMGS907.

### *mfa12345* locus

Significant E-values ranging from e^−200^ and e^−100^ were observed for each of the five Mfa12345 profiles (Fig. S3C). Specifically, regarding the Mfa1 HMM profile, three distinct situations were evident: (i) Mfa1 was recovered, with low E-values, in four species (*P. gingivalis*, *P. gulae*, *P. loveana* and *P. macacae*); (ii) in 14 groups, Mfa1 was identified with higher E-values; and (iii) in six species (*P. bennonis*, *P. canoris*, *P. catoniae, P. cangingivalis* and *P. pasteri*, as well as PSP_OH3588), no Mfa1 was detected. The Mfa2 HMM profile produces identical results, yielding the same three groups.

The Mfa3 HMM profile successfully identified this protein in the same four species (*P. gingivalis*, *P. gulae*, *P. loveana* and *P. macacae*) and additionally in *P. endodontalis* that contains an Mfa3-like protein. Finally, both the Mfa4 and Mfa5 HMM profiles exclusively detected these proteins in *P. gingivalis*, *P. gulae* and *P. loveana* and in three of the six strains of *P. macacae*: JCM15984 and NCTC11632 (isolated from the oral cavity of cats) and OH2859 (isolated from a canine oral cavity). In OH2859, the *mfa12345* operon locus is intact, while in JCM15984 and NCTC11632, we observed two distinct loci: the first one contains genes encoding Mfa123 proteins, followed by two genes encoding proteins similar to FimD and FimE (referred to as *mfa123_fimDE*), and the second comprises genes encoding Mfa2345 proteins preceded by a non-characterized fimbrilin gene that shares similarity with Ffp1, indicated by low E-values of 7.3e^−58^ for Ffp1 profile A and 7.8e^−41^ for Ffp1 profile B (referred to as *ffp1-like_mfa2345*). It is worth noting that three strains of * P. macacae*, specifically OH2631 (isolated from the canine oral cavity), as well as NCTC13100 and DSM20710/JCM13914 (isolated from the macaque oral cavity), exhibit two tandemly organized *mfa123_fimDE* loci. Remarkably, these loci are not identical, displaying an average sequence identity of 53 %. In OH2631, these loci are separated by less than 2 kb, while in NCTC13100 and DSM20710, they are separated by a 3 kb region that includes an IS4 pseudogene. None of these three strains harbour the *ffp1-like_mfa2345* locus.

*P. endodontalis* features an additional alternative locus comprising six genes, including Mfa1-like, Mfa2 and Mfa3-like, followed by two genes encoding lipoproteins and one gene encoding a von Willebrand factor type A (VWA) domain-containing protein. Interestingly, several other species, such as *P. asaccharolytica*, *P. circumdentaria*, *P. crevioricanis*, *P. gingivicanis* and *P. uenonis*, also exhibit alternative loci, which probably correspond to novel fimbrilin systems. These systems require in-depth dedicated future studies for thorough characterization.

In conclusion, when considering only the complete *mfa12345* locus as a reference, we identified its presence in four species: * P. gingivalis*, *P. gulae*, *P. loveana* and *P. macacae* strain OH2859. We also illustrate the effectiveness of HMM profiles in distinguishing true *mfa* loci from alternative loci. As for FimABCDE, the descriptions found in the annotations of Mfa12345 proteins are uninformative, often annotated as hypothetical or fimbria. This labelling makes it nearly impossible to conduct meaningful ontology searches (Fig. S3D).

### ffp1

MMseqs2 clustering reveals the separation of Ffp1 orthologues in two distinct groups which resulted in two distinct HMM profiles termed Ffp1_A and Ffp1_B (Fig. S3E). Ffp1_A mature amino acid sequences, excluding the signal peptide, share 57.4 % identity and 23 conserved amino acids (logo in Fig. S3E), while Ffp1_B sequences exhibit only 37 % identity primarily due to divergence in *P. bennonis,* but with 30 conserved amino acids (logo in Fig. S3E). The identity between the two groups decreases to 24 % with only eight conserved amino acids (represented with an asterisk in the logos Fig. S3E).

The Ffp1_A HMM profile retrieves genes from all *Porphyromonas* species except *P. bennonis*, *P. canoris*, *P. cangingivalis*, * P. levii* and *P. somerae*, which are recovered with the Ffp1_B HMM profile. So, remarkably, fimbrillin Ffp1 is indeed present in all *Porphyromonas* spp., contrary to FimABCDE and Mfa12345 [except for *P*. sp. UMGS1085 where a 186 nt fragment of a gene (at the start of a contig) is identified by the Ffp1_A HMM profile with an E-value at 6.7 e-22 (Fig. S3E)]. This higher E-value is the result of being obtained for only 61 amino acids instead of about 500 for an Ffp1_A protein.

As shown in figure Fig. S3F, approximately 70 % of the identified Ffp1 proteins are annotated as hypothetical or uncharacterized, 22 % as fimbrillin/fimbriae (with half linked to the PGN_1808 protein, described as Ffp1 in the *P. gingivalis* ATCC 33277 reference strain) and 8 % as lipoproteins.

Using HMMsearch with both Ffp1_A and Ffp1_B profiles, using an E-value threshold at e^−100^, in the Ensembl Genome Bacteria (taxid:2) database, only *Porphyromonas* proteins are retrieved. We conclude that Ffp1 fimbrillins are the sole fimbriae proteins conserved across all *Porphyromonas* species, making them unique to the genus.

## Characterization of *Porphyromonas* FFp1 fimbriae

**Fig. 3.**
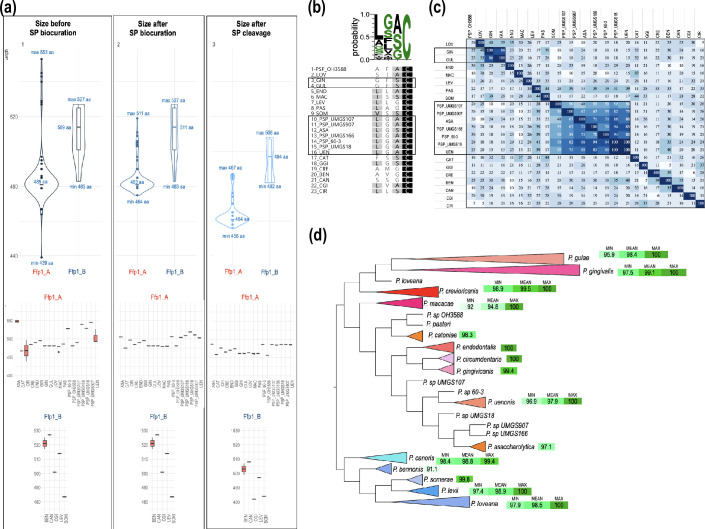
(a) Violin plots of Ffp1_A and Ffp1_B amino acid lengths. From left to right: sizes as initially annotated in GenBank files (no curation), sizes after signal peptide (SP) biocuration prior to cleavage and sizes after SP cleavage by signal peptidase II (SPII). In the box plot associated with each violin plot, the middle line represents the median and the whiskers indicate the interquartile range. (b) Multiple sequence alignment and sequence logo of Ffp1 lipobox. Boxes represent groups of identical sequences. (c) Heat map illustrating the percentage nucleotide identity of Ffp1 signal peptides. (d) Phylogram of *Porphyromonas* Ffp1 proteins distance tree. The Ffp1_A proteins are depicted in warm colours, while Ffp1_B proteins are shown in various shades of blue. The boxes indicate the minimum, average and maximum intraspecific identity values. If only one value is displayed, it represents the average identity percentage.

Ffp1 exhibits variable pre-cleavage sizes among *Porphyromonas* species, in both subclasses. For the Ffp1_A group, protein sizes range from 439 aa (*P. circumdentaria* DSM 103022) to 553 aa (*P. asaccharolytica* PR426713P-I), and for the Ffp2_B group, from 483 aa (*P. somerae* DSM 23387) to 527 aa (*P. canoris*) (Fig. 3a). Size is well conserved within *Porphyromonas* species except for * P. asaccharolytica*, *P. circumdentaria*, *P. macacae* and *P. uenonis* for Ffp1_A, and *P. bennonis* for Ffp1_B (Fig. 3a).

The observed differences for *P. asaccharolytica* are due to the presence of 33 additional nucleotides in strain PR426713P-I (at position 88–120), absent in strain DSM 20707. For *P. circumdentaria*, it is a 175 nt shorter annotation in strain DSM 103022 (compared to strain ATCC 51356). For *P. macacae* these are due to the gene encoding Ffp1_A being at the end of the contig and truncated at the 5′ end, in strain *P. macacae* JCM 15984. For *P. uenonis*, it is also the choice of an alternative start codon for the UMGS1452 strain, 34 aa upstream of those chosen for the DSM 23387 and JCM 13868 strains. Finally, for * P. bennonis*, at position 1410 in the DSM 23058 strain, a C base, absent from the JCM 16335 strain, leads to a frameshift. This frameshift leads to a shorter C-terminal sequence compared to DSM 23058. Note that for *P. somerae*, the sizes are similar, but the annotated sequences are ‘shifted’ and proteins different on the N-terminal (20 aa longer in DSM 23387 compared to St14) and C-terminal [21 aa shorter in DSM 23387 due to a partial coding sequence (CDS) at the end of the contig].

Accurate annotation of the N-terminus of proteins, which predicts their cellular localization, is crucial and deserves the attention of annotators. For this, we re-annotated the start codons of Ffp1, when needed, to optimize both the SPII cleavage prediction score and the presence of charged residues at the N-terminus, followed by hydrophobic amino acids. The resulting re-annotations and their implications for cell localization predictions are listed in Table S2.

In the absence of thorough human biocuration for structural annotation, particularly regarding the selection of start codons, a significant portion of Ffp1 proteins are predicted to be cytoplasmic (*P. asaccharolytica*, *P. catoniae*, *P. circumdentaria* DSM 103022, *P. somerae* St14) or having localization predictions classified as indeterminate (PSP UMGS107, PSP UMGS166, PSP UMGS907, * P. uenonis* DSM 23387, *P. uenonis* JCM 13868). Some proteins are predicted to be cleaved by SPII, but biocuration enhances both the signal peptide prediction score and the likelihood of cleavage by SPII. As a result of this reannotation work, all Ffp1 proteins are predicted as lipoproteins, with a signal peptide of about 20 aa (15–25 aa), consistent with the requirements cited previously: two to four positively charged amino acids followed by a hydrophobic region of 10–15 aa (Fig. S4) and a lipobox [ASG]↓C positions −1 to 1 (Fig. 3b). *In silico* predictions also confirm the predicted palmitoylation (addition of acyl chains) of the cysteine residue.

These biocurated peptide signals exhibit a high degree of intra-species conservation, while demonstrating significant inter-species variability, with only a 25 % pairwise identity when considering all species collectively (min. 5 %, max. 100 %; Fig. 3c). However, two groups characterized by similar signal peptide sequences can be discerned: a first one formed by * P. gingivalis* and *P. gulae* (ca. 86 % identity) and a second more consistent, composed of *P. asaccharolytica*, *P. uenonis*, PSP_60-3, PSP_UMGS907, PSP_UMGS18 and PSP_UMGS166 (66.7–100 % identity, Fig. 3c). The same groups were observed when examining the lipobox motif.

As shown in Fig. 3a (second panel), Ffp1 signal peptide biocuration not only results in more consistent predictions of their cellular localization, but also leads to a homogenization of their size, both within and across species, except for *P. bennonis* (since the frameshift occurs in the 3′ region of the gene). This size homogenization becomes even more pronounced following signal peptide cleavage (Fig. 3a, third frame). Mature Ffp1s in group B are larger than those in group A by about 20 aa.

As shown in Fig. 3d, the average intra-specific identity of the Ffp1_A subclass is very high and ranges from 100 % to 94.8 % depending on the species. The most divergent species are *P. macacae*, *P. gulae* and *P. uenonis*. In the first two cases, this divergence can be attributed to the coexistence of two distinct homology groups within the same species. However, regrettably, the available metadata do not provide sufficient information to elucidate the underlying reasons for these discrepancies. For *P. uenonis*, strain UMGS1452 derived from a metagenome is different from the two other strains. As previously noted, the conservation of interspecific Ffp1_A sequences is low (57.5 %) with only 4.5 % of identical sites between all of them. When examining the Ffp1_B group, it is worth noting that the average intra-specific identity is elevated, oscillating between 98.8 and 91.1 % (Fig. 3d). *P. bennonis* is the most divergent because the two strains have proteins with the last 75 aa that differ. It is noteworthy that Ffp1_B is less homogeneous than Ffp1_A with an average inter-specific identity of only 36.4 % and 4.3 % identical sites. The number of conserved sites decreases to 0.7 % if we compare both groups, Ffp1_A and Ffp1_B.

## 3D structures confirm that *Porphyromonas ffp1* are fimbrillins

As the signal peptide is absent in the mature protein, it was excised prior to structure prediction for all Ffp1 proteins. PSIPRED predicts 30–44 % residues as strand (mean=36.6, sd=3.4) and 2–7 % residues as helix (mean=5.4, sd=1.4) for the Ffp1_A group. For the Ffp1_B group, predictions concern 24 %–42 % amino acids as strand (mean=29.6, sd=6.5) and 4 %–9 % as helix (mean=7, sd=2.1).

**Fig. 4.**
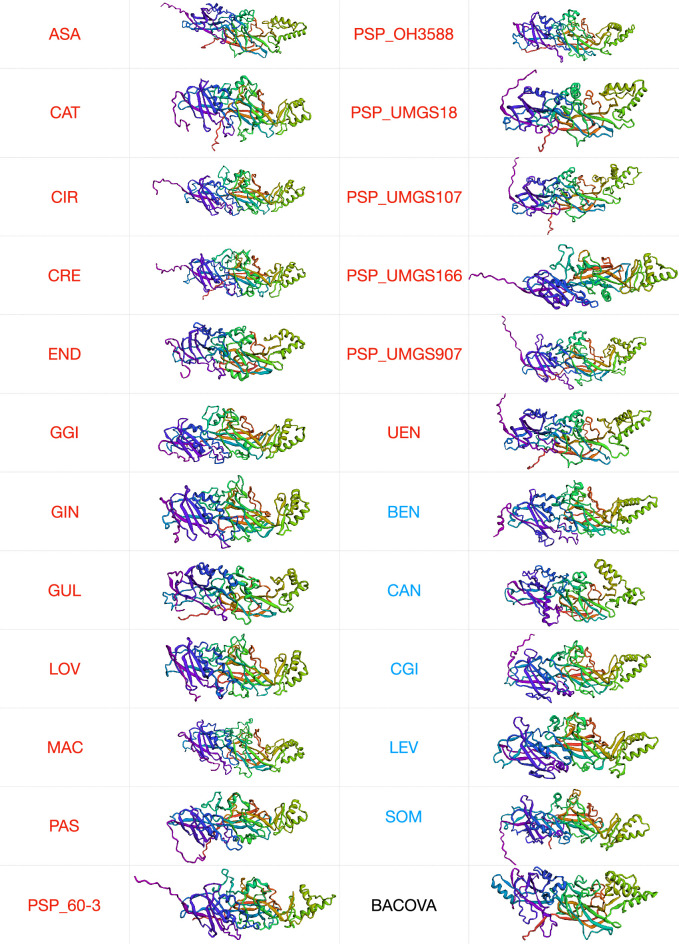
Predicted tertiary structure for mature proteins of *Porphyromonas* reference strains (one per genus). These structures correspond to predictions made by Robetta and evaluated by ERRAT and Verify3D. Only the best prediction is represented. Ffp1_A proteins are in red and Ffp1_B in blue. BACOVA_01548 was also predicted using Robetta.

The optimal structures for all *Porphyromonas* Ffp1 representatives, as predicted by Robetta and assessed by ERRAT and Verify3D, are depicted in Fig. 4. These structures were subjected to comparison with existing models and, the best hit, obtained either via VAST+ or Phyre2, corresponds to *Bacteroides ovatus* cell adhesion protein (BACOVA_01548, 4JRF.pdb) for all *Porphyromonas* Ffp1 proteins, irrespective of species or Ffp1_class.

According to Phyre2 and iBPA results (Fig. S5), more than 82 % of Ffp1 sequences were modelled with 100.0 % confidence against 4JRF.pdb. Superposition of *Porphyromonas* Ffp1 and BACOVA_01548 3D structures were performed by iBPA and all evaluation values (RMSD, GDT_TS) reflect good overall similarity. For all overlapping morphologies, the aligned fraction is about 50 % of the protein sequence, with mean reported RMSDs of 2.26 Å (range 2.09–2.53 Å) and mean GDT-TS distance scores of 32 (range 32–37.3 Å) (Fig. S5). For *P. gingivalis*, the structures of FimA (4Q98.pdb) and Mfa1(5NF2.pdb) are available and comparisons by superposition between Ffp1 and these two other fimbrillins (Fig. S6) confirm that Ffp1 is indeed a new distinct *Porphyromonas* fimbrillin family.

## *Porphyromonas* Ffp1 are ancestral orthologues but not syntelogues

In *P. gingivalis*, *ffp1* is the fourth gene in an operon-like structure comprising a gene encoding a cysteinyl-tRNA synthetase, a second gene encoding a patatin-like protein, and a third gene encoding a group 2 glycosyltransferase. An identical locus is found in all *P. gulae* genomes, while t is absent in all other *Porphyromonas* (Fig. S7). The *P. asaccharolytica*, *P. uenonis*, *P*. sp. UMGS18 and * P*. sp. UMGS107 group, mentioned above, show a syntenic pattern upstream of *ffp1*, characterized by the presence of two conserved genes encoding dihydroorotate dehydrogenases, crucial enzymes involved in *de novo* pyrimidine biosynthesis in prokaryotic cells. *P. uenonis* and *P.* sp*.* UMGS18 even extend this 5′ synteny with the gene *uvrA* encoding excinuclease ABC subunit A. *P. uenonis, P.* sp*.* UMGS18 and *P.* sp*.* 60-3 also share a *ffp1* downstream gene encoding a potassium/proton antiporter. *P. asaccharolytica*, * P*. sp. UMGS166 and *P*. sp. UMGS907 show three syntenic genes downstream of *ffp1*, one encoding a nitronate monooxygenase (degradation of propionate-3-nitronate), another encoding a 4-hydroxy-tetrahydrodipicolinate synthase (involved in lysine biosynthesis) and *recF*, involved in DNA replication and repair. Finally, *P. catoniae* and *P. pasteri* also share three conserved genes, upstream of *ffp1*, encoding respectively a serC phosphoserine transaminase, an NAD(P)-binding domain-containing protein and a protein with the DUF1015 domain (Fig. S7).

However, linking all these surrounding gene functions with *ffp1* fimbrillin is challenging, if not impossible, without functional experimentation. Furthermore, the intergenic spaces, often spanning several hundred nucleotides, suggest separate regulatory mechanisms and rule out any functional correlation between these genes. For the other *Porphyromonas* species, each exhibits a distinct gene organization arrangement surrounding *ffp1* (Fig. S7).

**Fig. 5.**
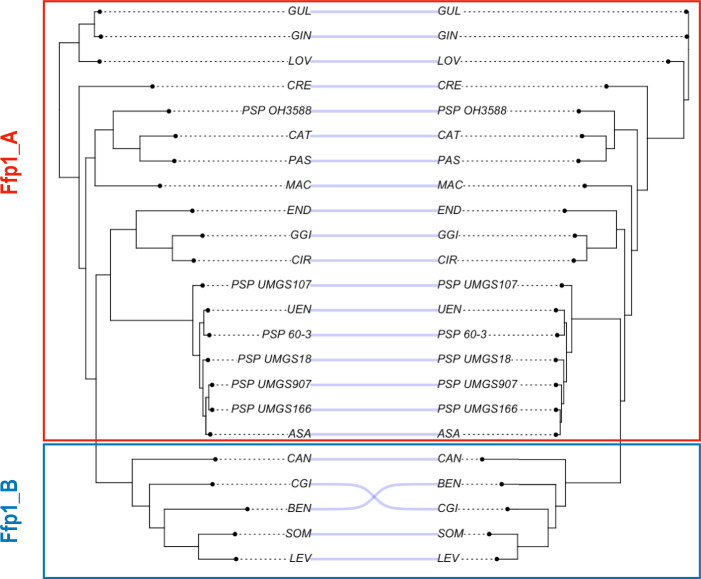
Tanglegram comparing the tree reconstructed from the primary sequences of the Ffp1 proteins in representative strains of *Porphyromonas* (on the left) with the species tree based on the orthology of the orfeomes.

In conclusion, except for phylogenetically closely related species, we find no preserved synteny in the *ffp1* locus, which would reflect the absence of co-localization constraints for co-functional genes. Nevertheless, as demonstrated by the tanglegram juxtaposing the orfeome tree and the Ffp1 tree (Fig. 5), the remarkable congruence between these two trees provides compelling evidence that Ffp1 is an ancestral protein of *Porphyromonas,* and its evolution would have closely paralleled the evolutionary trajectory of the entire genus. This observation also holds true for the differentiation between the two Ffp1 classes (Fig. 5). The absence of gene conservation in close chromosomal proximity to *ffp1,* along with the presence of a significant 5′ intergenic space (Fig. S7), not only signifies the absence of selection pressure around this gene but also strongly suggests that *ffp1* functions as an independent transcriptional unit. These results suggest strict vertical inheritance of *ffp1* in the genus *Porphyromonas* over a long period of evolutionary time, demonstrating that this fimbrillin is part of the *Porphyromonas* pangenome and is not an accessory gene. However, it should be noted that this locus appears to have evolved differently in each species or related group of species, as no strict synteny is observed.

## Discussion

Our analysis of fimbrillin loci within the genus *Porphyromonas* was initiated with the retrieval of genomes from the NCBI RefSeq database. The first step encompassed the validation of genus-level assignment for each genome retrieved, followed, when feasible, by species-level confirmation. The overall genome relatedness indices (OGRIs), namely digital DDH distance and gANI, were used to classify genomes into monophyletic groups. These OGRIs are increasingly used in taxogenomic studies and serve as a valuable tool for validating the taxonomic classification of isolates of interest [84]. Likewise, in accordance with prior research, we employed more conventional methodologies for species-level genome grouping, such as evaluating the percentage identity of the gene encoding 16S rRNA (when annotated) [85].Our study underscores the critical necessity of rigorously confirming the taxonomic classifications of genomes before embarking on any comparative genomics analysis to ensure their accuracy. Moreover, this checking step enables the possibility of taxonomic reassignment when warranted, aligning with findings from previous studies [86,88]. In this investigation, we have identified genomes erroneously labelled as *Porphyromonas* (i.e strain 31_2, which is a *Parabacteroides*), misassignment of *Porphyromonas* to species (i.e strain 60.3, which does not belong to *P. uenonis*) as well as metagenomic mixture such as strain KA000683 imperfectly assigned to *P. somerae*.

Our study also raises questions about genomes assigned to *Porphyromonas* without any species assignment (28 out of 144 genomes, i.e. 19.5 %). They all correspond to incomplete draft genomes which introduces bias into studies that rely on them [89]. We specifically note the presence of gaps, local assembly errors, chimeras and contamination by fragments from other genomes [90,91]. This contamination, defined as the presence of foreign sequences within a genome, can lead to incorrect functional inferences such as higher rates of horizontal gene transfer (HGT) and errors in phylogenomic studies. Such errors can be propagated throughout the scientific community and have been documented to exist in databases [91]. To mitigate these types of errors, several studies, including the present one, advocate the practice of data biocuration throughout the study. To identify potential contamination in draft genomes, we employed Kraken2 software and assessed the cumulative contig size of incomplete genomes. By applying specific inclusion criteria, we were able to disqualify 17 draft genomes, corresponding to metagenomic mixtures and inaccurately labelled as *Porphyromonas*. Furthermore, among the 11 remaining draft genomes, our taxogenomic approach led to the reclassification of five genomes into four previously described species (*P. gulae*, *P. asaccharolytica*, *P. uenonis* and two genomes in *P. canoris*). The remaining six genomes that cannot be assigned to already described species may potentially represent novel, yet undescribed species, akin to hypotheses proposed in other bacterial genera [88,92]. This suggests that the genus *Porphyromonas* may encompass a greater degree of species diversity than previously recognized.

Thus, in this study, we retained 126 *Porphyromonas* genomes (24 clades comprising 17 species and seven singletons) to describe fimbriae loci. To accomplish our research objectives, distant homology between proteins must be detected and is fundamental for enabling comparative and evolutionary investigations, shedding light on protein families, and providing insights into their molecular structures and functions [93].

Current orthology detection methods include position-specific scoring matrix (PSSM) techniques, like PSI-blast (position-specific iterated blast [94], which generate substitution score profiles by accounting for residue variability within homologous sequence families [95]. An even more effective approach involves HMM profiles, which incorporate emission and transition state probabilities at each protein sequence position, making them a superior choice for identifying distant homology [95,96].

Using ontology as a protein search strategy search is ineffective, as most fimbrillin genes are poorly annotated or annotated as ‘hypothetical protein’ (between 21.1 and 88.6 % of annotated genes). Specifically, stem and anchor proteins (FimAB or Mfa12) are better annotated with deficient annotation rates ranging from 21.1 to 50.7 %. In contrast, accessory proteins (FimCDE or Mfa345) suffer from particularly poor annotations with error percentages ranging from 58.9 to 88.6 %. These annotation errors are present within the databases and, without biocuration and correction, are likely to persist, potentially perpetuating inconsistencies, inaccuracies and errors in subsequent genome annotations [97]. For example, for a gene family, nearly 20 % of sequences may exhibit significant errors such as inaccuracies in gene names, partial sequences or initiation codon misassignments [98]. In the context of less extensively researched bacterial species, as is the case in this study, the prevalence of erroneous or uninformative annotations are much higher, reaching 77.1 % of sequences identified as Ffp1 where the annotation was ‘hypothetical protein’ or ‘lipoprotein’.

In this study, we utilized 12 HMM profiles developed from *P. gingivalis* genomes, which were further refined through a strategy involving functional domain screening, clustering and biocuration. This approach enabled a comprehensive exploration of the *Porphyromonas* orfeomes, revealing variations in the three fimbriae loci across all species within this genus.

The *fimABCDE* locus is present in nine (of 24 groups, or 37.5 % of *Porphyromonas* species) with two distinct *fim* loci present in all *P. macacae* genomes. The *mfa12345* locus is present only in three closely related species (*P. gingivalis*, *P. gulae* and *P. loveana*). For this locus, hybrid *fim/mfa* or *ffp1/mfa* loci are present in two species (*P. endodontalis* and *P. macacae*): *mfa123_fimDE* and *ffp1-like_mfa2345* in *P. macacae*; and a distinctive six-gene locus in *P. endodontalis*. This locus encompasses genes encoding Mfa1-like, Mfa2 and Mfa3-like proteins, along with two genes responsible for lipoproteins and a gene encoding a protein featuring a VWA domain. Interestingly, for the gene encoding Mfa5, the prevailing description is rather nondescript, simply stating it as a ‘protein containing a VWA domain’. This description, however, falls short in conveying the functional significance of this gene. It is worth emphasizing that proteins featuring VWA domains play pivotal roles in diverse biological processes, including but not limited to cell adhesion and defence mechanisms. Thus, a more detailed annotation is warranted to better appreciate the functional implications of Mfa5 [99].

Finally, other species (i.e. *P. asaccharolytica*, *P. circumdentaria*, *P. crevioricanis*, *P. gingivicanis* and *P. uenonis*) have fimbrilin genes identified through HMM profiles that remain uncharacterized. These two loci, *fimABDCE* and *mfa12345*, have been described in other closely related species, for example an Mfa system (with only *mfa1* and *mfa2*) in *Bacteroides thetaiotaomicron* [100], and a cluster with *fimABCDE*-like genes and genes similar to either *mfa1*/*mfa2* or *mfa4*/*mfa2* with either *mfa1* or *mfa4* encoding the fimbriae stem and *mfa2* as an anchor in *Parabacteroides distasonis* [101]. The *fim* and *mfa loci* in *Porphyromonas* spp. will be the main subject of an further publication.

Concerning Ffp1 fimbriae (77.1 % of all *ffp1* genes were deficiently annotated), this protein was most recently described in *P. gingivalis* [14,15]. The encoding gene has two variants, denoted as A and B in our study. Ffp1_A is the predominant variant found in 19 *Porphyromonas* species/groups, whereas Ffp1_B is restricted to only five species (*P. bennonis*, *P. canoris*, *P. cangingivalis*, *P. levii* and *P. somerae*). Furthermore, this study demonstrates that the utilization of HMM profiles reveals that *ffp1* is confined to the genus *Porphyromonas* and is absent in closely related genera such as *Bacteroides* or *Prevotella*. This finding contrasts with approaches employing blastp and PSI-blast [17].

In the future, for certain *Porphyromonas* species with limited genomic data, it will be important to revalidate the absence of * fim* and/or *mfa* fimbrillin loci through extensive genome sequencing efforts, particularly targeting several strains. In comparative genomics, the inherent incompleteness of draft genomes demands careful consideration to nuance results, particularly when the conclusions concern gene absence. Nevertheless, in our investigation, absence of the *fim* and/or *mfa* locus was substantiated by the non-detection of all ten corresponding genes, which, we believe, accentuate the robustness of our findings. In a broader sense, it is regrettable that raw reads are not available for download for most draft genomes. Having access to these raw reads could potentially allow for the confirmation of gene absence when needed.

The presence of multiple fimbriae loci within genomes is a common phenomenon observed in other bacterial models. These loci are often associated with general niche colonization abilities or the adhesion to more specific substrates [102,103]. Further investigations are needed on species more closely related to *Porphyromonas* and within this bacterial genus. These studies can shed light on aspects such as host specificity and their association with species-related pathologies [104].

Given that the majority of *in silico* CDS annotators tend to prioritize the prediction of the longest possible ORF by favouring the initiation codon (ATG) over alternative codons (TTG and GTG) [105,106], and considering the variable size of proteins across *Porphyromonas* species, we conducted a thorough examination of the annotated initiation codons for each predicted Ffp1. Given that fimbrillins are lipoproteins [10], their N-terminal region is expected to feature a signal peptide starting with positively charged amino acids, followed by hydrophobic amino acids, and concluding with a cysteine-terminated lipobox, which serves as the cleavage site for SPII. The biocuration of start codons led to a more consistent protein size post-signal peptide cleavage. Additionally, the extracellular prediction of mature lipoproteins was confirmed, characterized by the presence of charged and hydrophobic residues, the lipobox, and a palmitoylation site. These features align with the ancestral nature of FFp1.

In addition, Ffp1 3D modelling of the mature protein was performed with several software packages, and the predictions were evaluated with classical metrics [107,108]. In all cases, the generated models were compared with existing 3D structures, and the most significant match was found with the cell adhesion protein BACOVA_01548 from *Bacteroides ovatus* [4]. This *B. ovatus* protein has not been extensively studied but was classified by the authors as the stem of a type V pilus, sharing common features with type V fimbriae. These characteristics include export to the periplasm as a lipoprotein (prepilin), subsequent delivery to the outer membrane, translocation to the cell surface and cleavage by Rgp (Arg-gingipain) [5,109].

Moreover, this new fimbrillin, Ffp1, exhibits notable distinctions from both FimA and Mfa1, as evident from the obtained metrics when superimposing the 3D structures of these proteins available for *P. gingivalis*. Furthermore, the gene arrangement of *ffp1* differs from the *fim* and *mfa* operons as the gene encoding Ffp1 does not appear to be in an operon structure. The strict vertical inheritance of *ffp1* in *Porphyromonas* suggests a vital role for this fimbrillin, as Ffp1 plays a role in responding to environmental signals, such as acid stress, and in polymicrobial biofilm production [14]. Moreover, it has been identified as enriched in sphingolipid-containing OMVs [18,19]. Thus, Ffp1 appears to serve crucial and diverse functions, facilitating *Porphyromonas* host colonization by promoting stress adaptation, biofilm formation and OMV production. The significance of these functions probably explains its vertical transmission and conservation within the genus *Porphyromonas*.

## Concluding remarks

HMM profiles are potent tools for detecting distant homologies and facilitating phylogenetic studies. For conducting these investigations, meticulous manual biocuration is essential, as with any *in silico* research. In this article, these HMM profiles make it possible to discriminate, without ambiguity, three *Porphyromonas* fimbriae and to describe their distribution: *mfa12345*, limited to the three closely related species (*P. gingivalis*, *P. gulae* and *P. loveana*); *fimABCDE* present in nearly 40 % of the *Porphyromonas* species; and *ffp1*, present in all *Porphyromonas* but restricted to this bacterial genus. Our study predicts that Ffp1 is a new fimbrillin, distinct from FimA and Mfa1. It is closely related to another type V fimbrillin protein, BACOVA_01548, as evidenced by manual start codon curation and 3D modelling. Given the ancestral nature of Ffp1, as elucidated by our study, and its presence in all studied *Porphyromonas* genomes, in contrast to the fimbrillins Fim and Mfa, the question of its function becomes paramount, especially in the absence of co-localization of accessory genes ensuring its stability, assembly and anchorage to the cell surface. What role does it play in the production and cargo of OMVs, a phenomenon observed in numerous studies? Further wet-lab investigations are necessary to address these pending inquiries.

## supplementary material

10.1099/acmi.0.000771.v3 Uncited Supplementary Material 1.

## Abbreviations

AF: alignment fraction
CDS: coding sequence
CU: chaperone-usher
DDH: DNA–DNA hybridization
gANI: genome average nucelotide identity
HGT: horizontal gene transfer
HMM: hidden Markov model
MAG: metagenome-assembled genome
OGRIs: overall genome relatedness indices
OMV: outer membrane vesicle
PSI-BLAST: position-specific iterated BLAST
PSSM: position-specific scoring matrix
SL: sphingolipid
SPII: signal peptidase II
VWA: von Willebrand factor type A
