# Supplementary material for: In silico analysis of Ffp1, an ancestral Porphyromonas spp. fimbrillin, shows differences with Fim and Mfa
Source: Access Microbiol. 2024 Jul 11;6(7):000771.v3. doi: 10.1099/acmi.0.000771.v3 (PMC11316588; doi:10.1099/acmi.0.000771.v3)
Supplement: Uncited Supplementary Material 1. [file acmi-6-00771-s001.pdf]

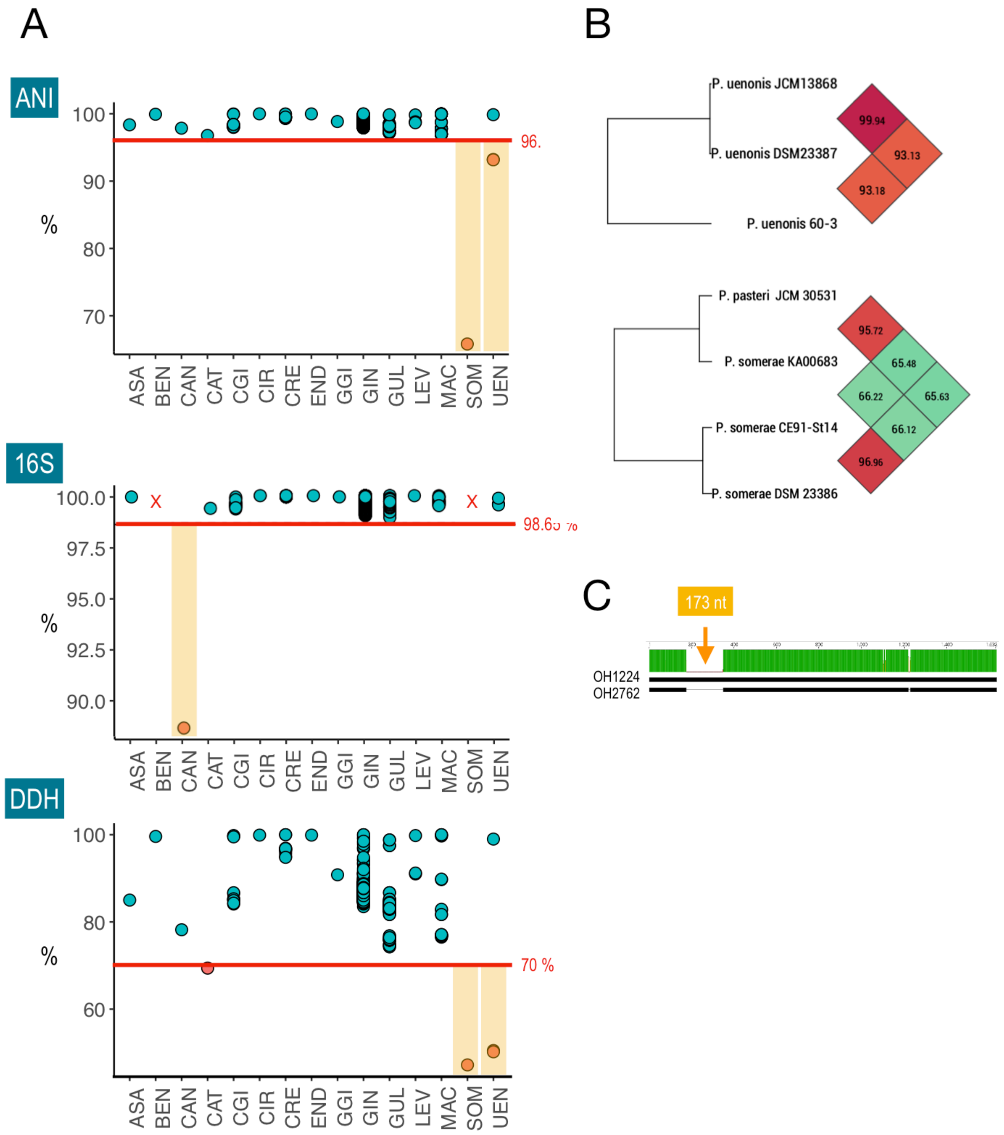

Figure S1. Validation of the taxogenomic assignment of *Porphyromonas* genomes. **A.** Intra-species homogeneity was checked by calculating intra-species distances using gANI, rRNA 16S identity and DDH. **B.** Checking *P. uenonis* and *P. somerae* genomes homogeneity using OrthoANI. **C.** Difference in 16S rRNA sequences of two strains of *P. canoris* with 173 nt insertion in strain OH1224. 3-letter code acronyms correspond to ASA: *P. asaccharolytica*; BEN: *P. bennonis*; CAN: *P. canoris*; CAT: *P. catoniae*; CGI: *P. cangingivalis*; CIR: *P. circumdentaria*; CRE: *P. crevioricanis*; END: *P. endodontalis*; GGI: *P. gingivicanis*; GIN: *P. gingivalis*; GUL: *P. gulae*; LEV: *P. levii*; LOV: *P. loveana*; MAC: *P. macacae*; PAS: *P. pasteri*; SOM: *P. somerae*; and UEN: *P. uenonis*.

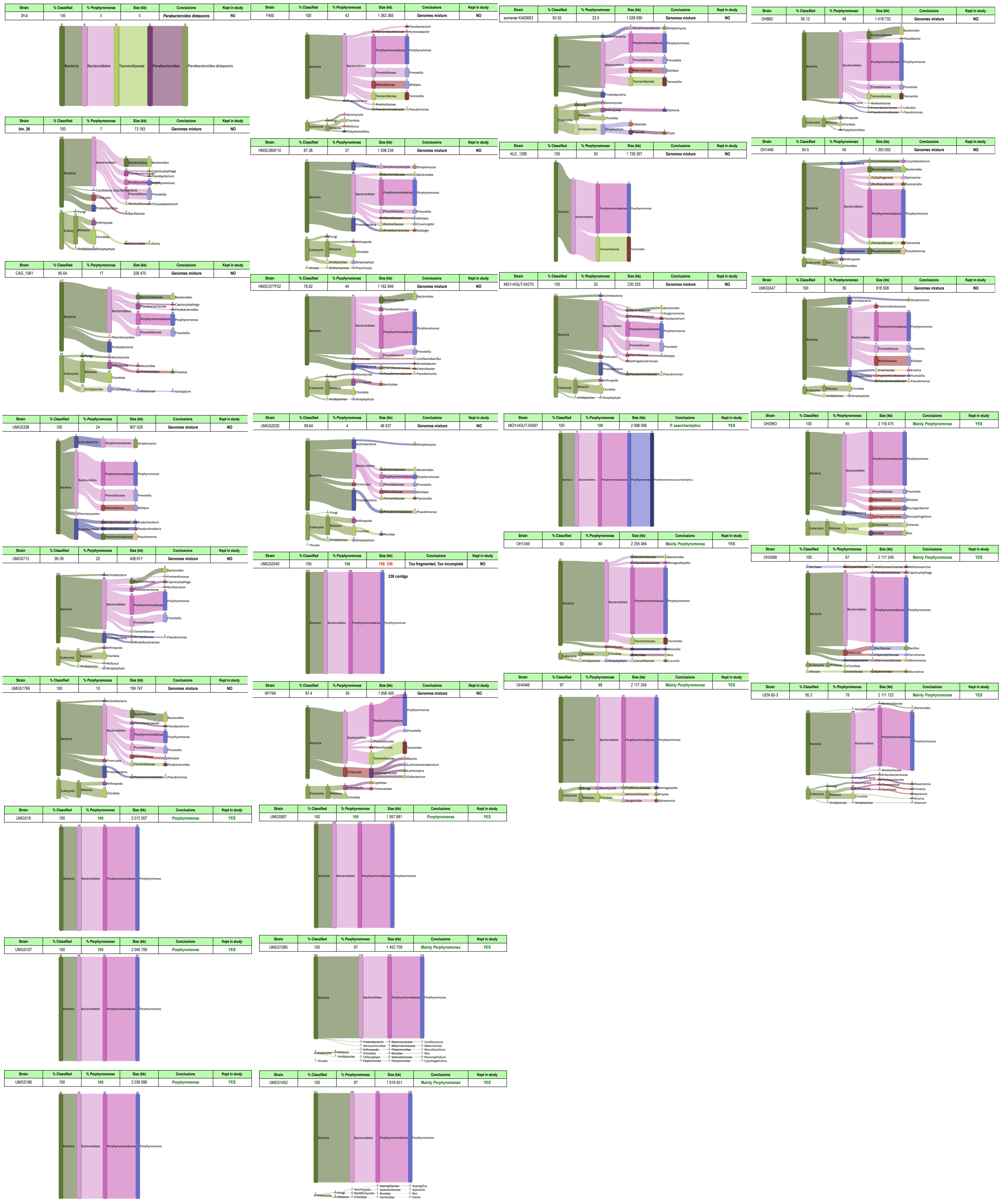

Figure S2. Sankey diagrams representing Kraken 2 report results for each *Porphyromonas* sp. genome. Each diagram illustrates the percentage of the genome classified under the genus *Porphyromonas* and the cumulative size of the accurately assigned fragments for each strain.

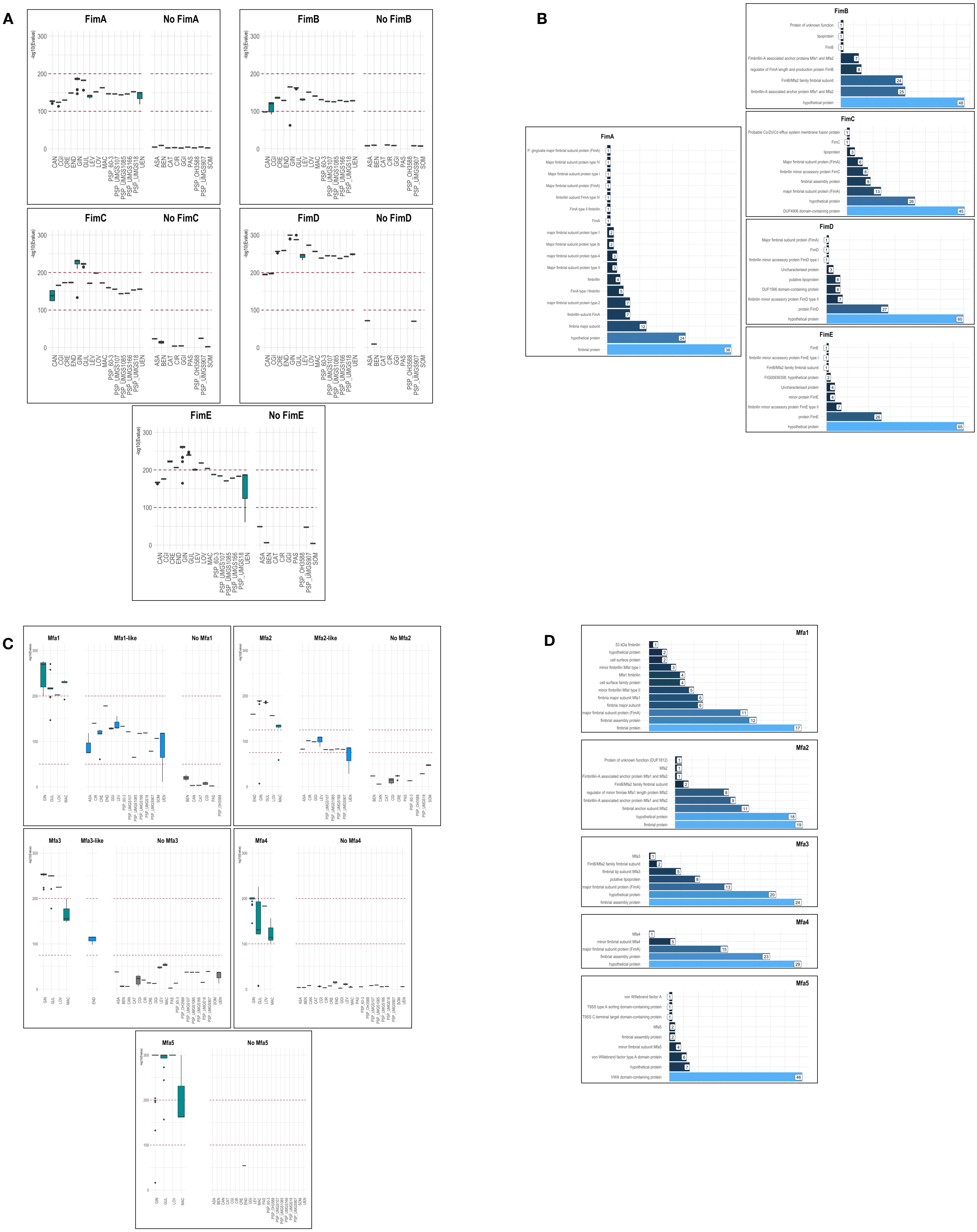
A
FimA
No FimA
FimB
No FimB
FimC
No FimC
FimD
No FimD
FimE
No FimE
B
FimA
FimB
FimC
FimD
FimE
C
Mfa1
Mfa1-like
No Mfa1
Mfa2
Mfa2-like
No Mfa2
Mfa3
Mfa3-like
No Mfa3
Mfa4
No Mfa4
Mfa5
No Mfa5
D
Mfa1
Mfa2
Mfa3
Mfa4
Mfa5

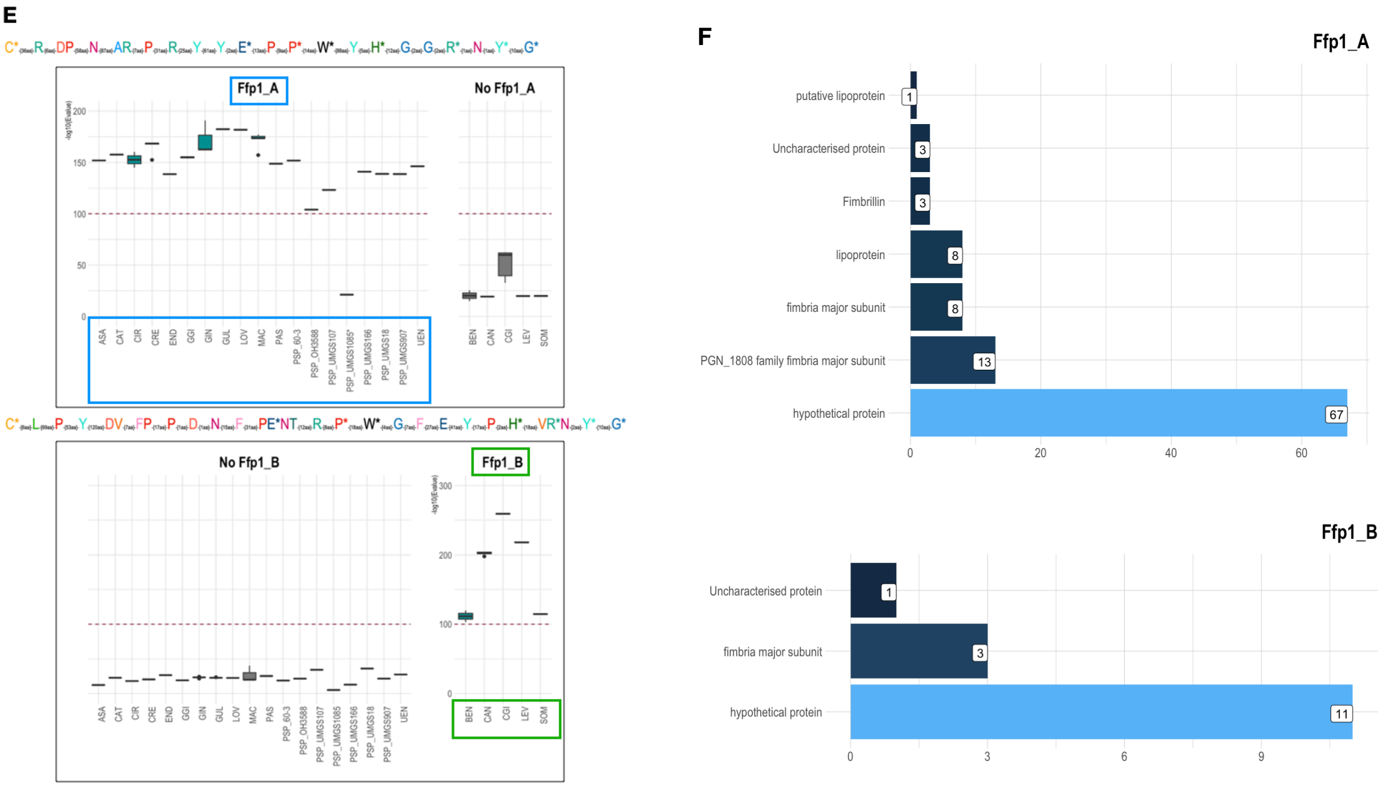

Figure S3. A-C-E: Box plot representing hmmsearch E-value results for all *Porphyromonas* genus groups and all HMM profiles used in this study**.** The dotted lines represent the thresholds used. In **E**, logo representation was used for Ffp1_A and Ffp1_B, capital letters are used for conserved amino acid position within each class (either A or B), and an asterisk denotes conserved positions in both classes. **B-D-F:** Frequencies of the terms employed to describe the genes of the 3 fimbriae systems of *Porphyromonas*, as originally annotated in the Genbank files.

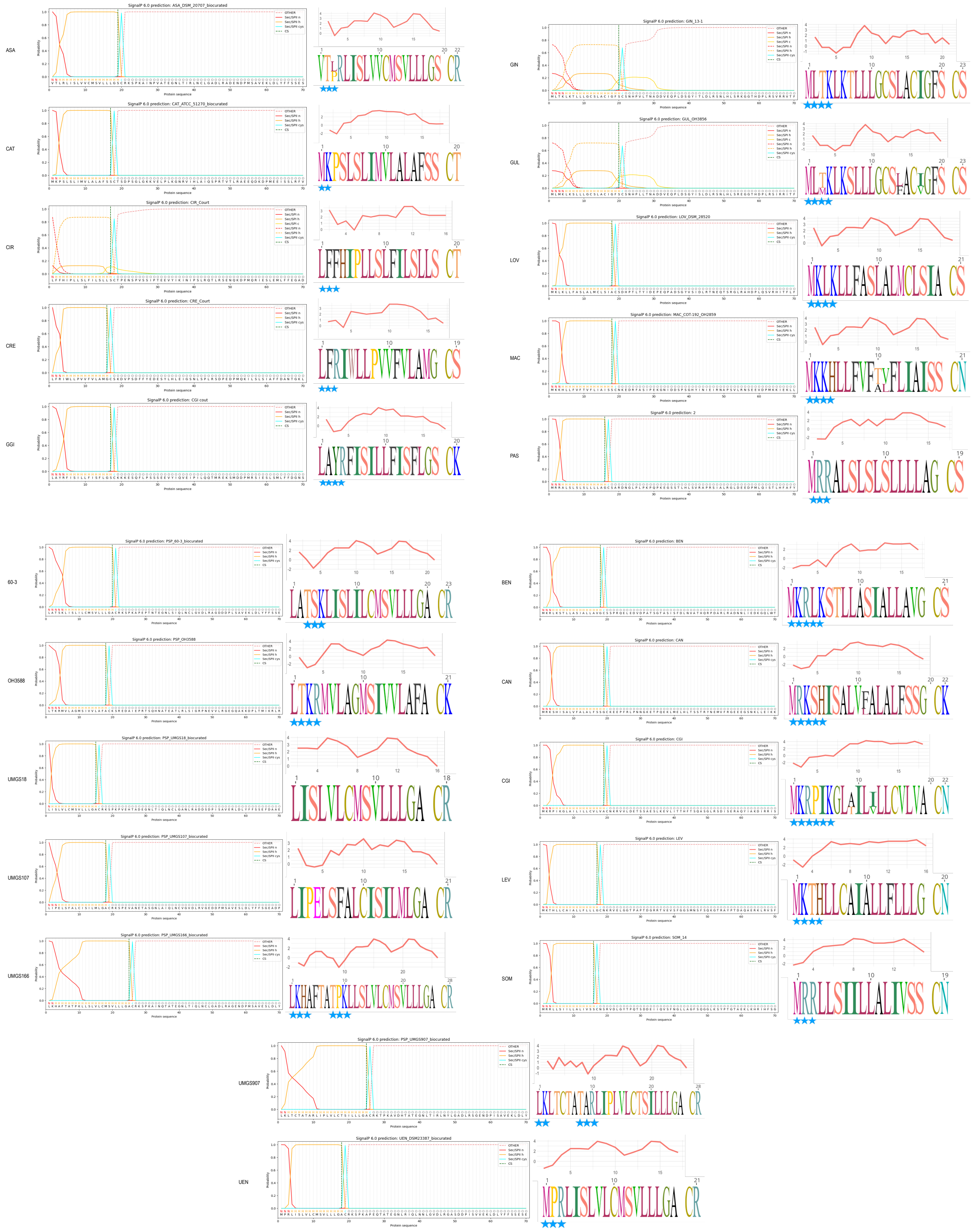

- **Sec/SPII**: lipoprotein signal peptides transported by the Sec translocon and cleaved by Signal Peptidase II (Lsp)

Figure S4. Ffp1 signal peptide prediction by the SignalP v6.0. For each *Porphyromonas* group, a reference sequence was chosen (named at the top of the SignalP graphs). For each group, we present the intra-specific consensus sequence of the signal peptides (after biocuration when required) in the form of a logo. The blue stars represent the charged amino acids (predicted by EMBOSS charge) and the orange curve represents the prediction of hydrophobicity (Protscale, Amino acid Hydropathicity using Kyte and Doolittle method).

Group	iPBA results	Phyre2 results	Superposition of predicted structure (green) with model structure (c4jrfA in red) using iPBA
ASA	Normalized score 90.90<br>RMSD 2.27<br>Alignment length 653<br>Aligned residues 339<br>Fraction aligned 51.91 %<br>GDT TS 33.25	430 residues (88% of sequence) have been modelled with 100.0% confidence	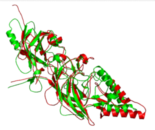
CAT	Normalized score 122.53<br>RMSD 2.09<br>Alignment length 622<br>Aligned residues 341<br>Fraction aligned 54.82 %<br>GDT TS 36.58	402 residues (88% of sequence) have been modelled with 100.0%	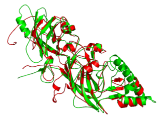
CIR	Normalized score 58.01<br>RMSD 2.25<br>Alignment length 631<br>Aligned residues 350<br>Fraction aligned 55.47 %<br>GDT TS 35.97	424 residues (89% of sequence) have been modelled with 100.0% confidence	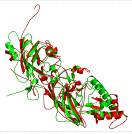
CRE	Normalized score 50.92<br>RMSD 2.42<br>Alignment length 636<br>Aligned residues 334<br>Fraction aligned 52.52 %<br>GDT TS 31.56	418 residues (90% of sequence) have been modelled with 100.0% confidence	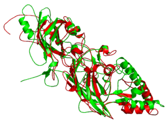
END	Normalized score 57.54<br>RMSD 2.18<br>Alignment length 636<br>Aligned residues 339<br>Fraction aligned 53.30 %<br>GDT TS 35.29	424 residues (90% of sequence) have been modelled with 100.0% confidence	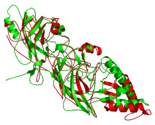
GGI	Normalized score 131.46<br>RMSD 2.13<br>Alignment length 629<br>Aligned residues 350<br>Fraction aligned 55.64 %<br>GDT TS 37.30	427 residues (90% of sequence) have been modelled with 100.0% confidence	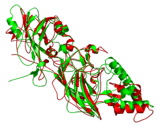
GIN	Normalized score 57.00<br>RMSD 2.20<br>Alignment length 637<br>Aligned residues 329<br>Fraction aligned 51.65 %<br>GDT TS 33.69	413 residues (90% of sequence) have been modelled with 100.0% confidence	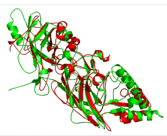
GUL	Normalized score 53.22<br>RMSD 2.20<br>Alignment length 647<br>Aligned residues 319<br>Fraction aligned 49.30 %<br>GDT TS 32.46	412 residues (90% of sequence) have been modelled with 100.0% confidence	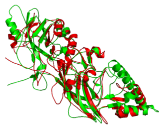
LOV	Normalized score 65.21<br>RMSD 2.27<br>Alignment length 659<br>Aligned residues 309<br>Fraction aligned 46.89 %<br>GDT TS 29.68	413 residues (89% of sequence) have been modelled with 100.0% confidence	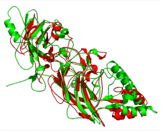
MAC	Normalized score 27.70<br>RMSD 2.27<br>Alignment length 635<br>Aligned residues 331<br>Fraction aligned 52.13 %<br>GDT TS 33.33	411 residues (89% of sequence) have been modelled with 100.0% confidence	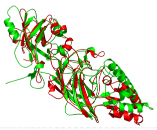
PAS	Normalized score 113.17<br>RMSD 2.29<br>Alignment length 636<br>Aligned residues 329<br>Fraction aligned 51.73 %<br>GDT TS 32.58	402 residues (88% of sequence) have been modelled with 100.0% confidence	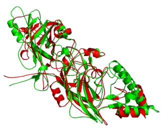
'SP_60-3	Normalized score 108.84<br>RMSD 2.24<br>Alignment length 657<br>Aligned residues 334<br>Fraction aligned 50.84 %<br>GDT TS 32.83	430 residues (89% of sequence) have been modelled with 100.0% confidence	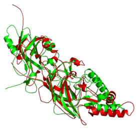

Group	iPBA results	Phyre2 results	Superposition of predicted structure (green) with model structure (c4jrfA in red) using iPBA
PSP_OH3588	Normalized score 57.31<br>RMSD 2.28<br>Alignment length 634<br>Aligned residues 327<br>Fraction aligned 51.58 %<br>GDT TS 32.72	403 residues (89% of sequence) have been modelled with 100.0% confidence	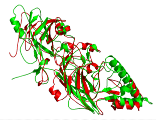
PSP_UMGS18	Normalized score 27.91<br>RMSD 2.22<br>Alignment length 663<br>Aligned residues 328<br>Fraction aligned 49.47 %<br>GDT TS 32.00	434 residues (89% of sequence) have been modelled with 100.0% confidence	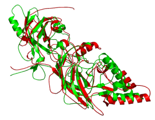
PSP_UMGS107	Normalized score 58.68<br>RMSD 2.22<br>Alignment length 654<br>Aligned residues 337<br>Fraction aligned 51.53 %<br>GDT TS 33.07	435 residues (90% of sequence) have been modelled with 100.0% confidence	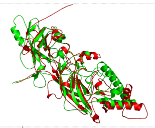
PSP_UMGS166	Normalized score 83.53<br>RMSD 2.28<br>Alignment length 657<br>Aligned residues 332<br>Fraction aligned 50.53 %<br>GDT TS 32.02	430 residues (89% of sequence) have been modelled with 100.0% confidence	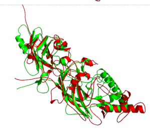
PSP_UMGS907	Normalized score 71.84<br>RMSD 2.19<br>Alignment length 664<br>Aligned residues 327<br>Fraction aligned 49.25 %<br>GDT TS 31.97	431 residues (89% of sequence) have been modelled with 100.0% confidence	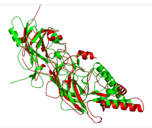
UEN	Normalized score -109.10<br>RMSD 2.39<br>Alignment length 668<br>Aligned residues 322<br>Fraction aligned 48.20 %<br>GDT TS 29.76	429 residues (89% of sequence) have been modelled with 100.0% confidence	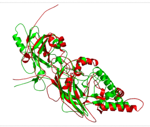
BEN	Normalized score 66.46<br>RMSD 2.25<br>Alignment length 682<br>Aligned residues 331<br>Fraction aligned 48.53 %<br>GDT TS 31.24	438 residues (86% of sequence) have been modelled with 100.0% confidence	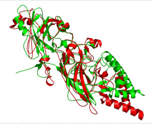
CAN	Normalized score 31.90<br>RMSD 2.28<br>Alignment length 677<br>Aligned residues 336<br>Fraction aligned 49.63 %<br>GDT TS 31.45	392 residues (77% of sequence) have been modelled with 98.9% confidence	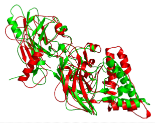
CGI	Normalized score -3.48<br>RMSD 2.35<br>Alignment length 640<br>Aligned residues 347<br>Fraction aligned 54.22 %<br>GDT TS 33.63	421 residues (88% of your sequence) have been modelled with 100.0% confidence	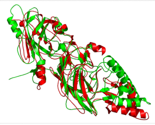
LEV	Normalized score 60.67<br>RMSD 2.53<br>Alignment length 673<br>Aligned residues 329<br>Fraction aligned 48.89 %<br>GDT TS 28.77	409 residues (82% of sequence) have been modelled with 100.0% confidence	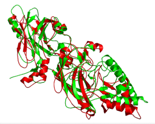
SOM	Normalized score 59.26<br>RMSD 2.37<br>Alignment length 647<br>Aligned residues 347<br>Fraction aligned 53.63 %<br>GDT TS 33.14	186 residues (38% of sequence) have been modelled with 100.0% confidence	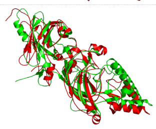

Figure S5. Evaluation of 3D models of Ffp1 proteins predicted *in silico*. Assessments were performed using iBPA and Phyre2 based on template 4JRF (BACOVA_01548). Right column shows superposition of predicted structure (green) with model structure (in red) using iPBA.

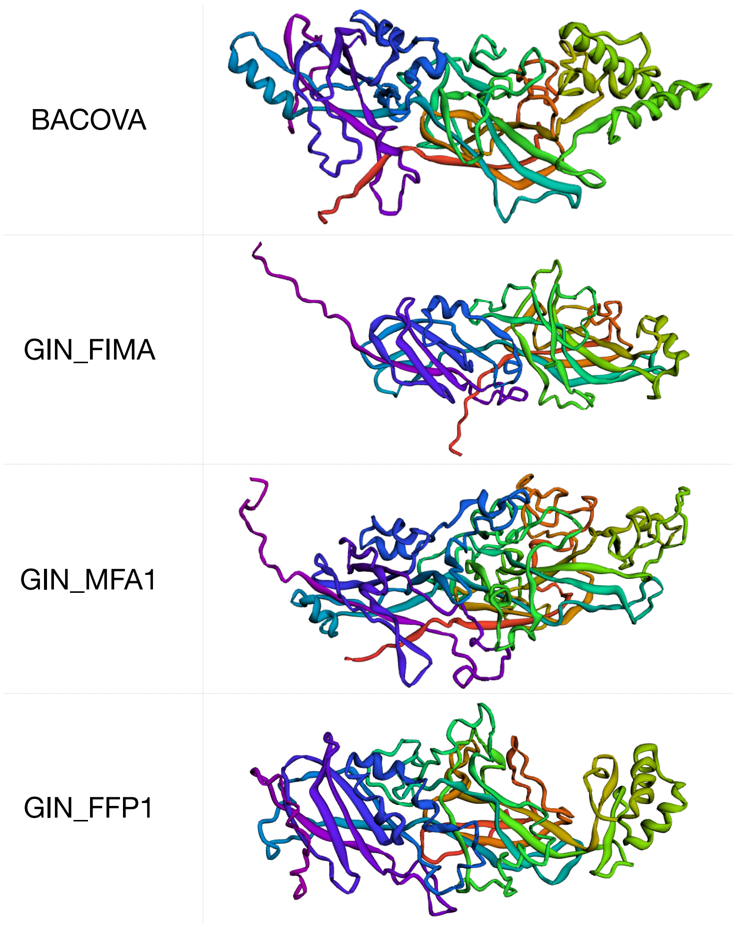

GIN_FFP1/BACOVA

Normalized score	57.00
RMSD	2.20
Alignment length	637
Aligned residues	329
Fraction aligned	51.65 %
GDT TS	33.69

GIN_FFP1/FIMA

Normalized score	-23.34
RMSD	2.48
Alignment length	588
Aligned residues	243
Fraction aligned	41.33 %
GDT TS	24.56

GIN_FFP1/MFA1

Normalized score	18.18
RMSD	2.17
Alignment length	645
Aligned residues	321
Fraction aligned	49.77 %
GDT TS	32.68

Figure S6. Comparison of Robetta structures prediction for *P. gingivalis* FimA, Mfa1 and Ffp1. The boxes represent the results of the superposition in Ffp1 and either BACOVA_01548, GIN_FimA or GIN_Mfa1.

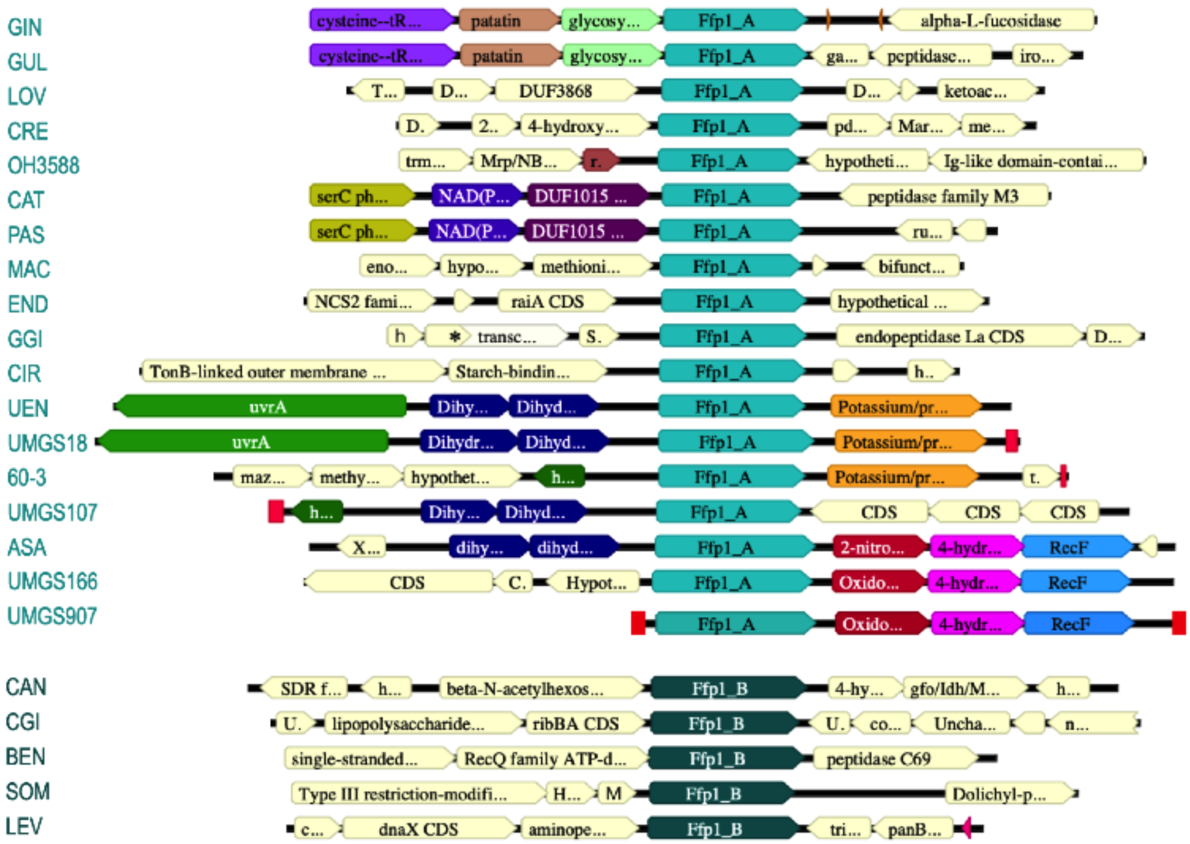

Figure S7. Diagram of synteny in the vicinity of the *ffp1* gene in the different species of *Porphyromonas*. Ffp1 genes are shown in turquoise (light for *ffp1_A* and dark for *ffp1_B*) and surrounding genes in the same color when synteny was conserved. When the surrounding genes are different, pale yellow was used. The diagram was made using Geneious Prime. The red boxes correspond to contig extremities.

List of *Porphyromonas* (and related information) used in this study

Species	Ref	Strain	Group acronym (this study)	Acronym (this study)	Genome Status (nb contigs)	Accession (WGS record)
asaccharolytica		PR42 6713P-I	ASA	ASA_PR426713P-I	Draft (58)	AENO00000000
asaccharolytica	X	DSM 20707	ASA	ASA_DSM 20707	Complete (1)	NC_015501.1
sp.		MGYG-HGUT-04267	ASA	ASA_MGYG-HGUT-04267	Draft (74)	CABPCR010000001-07
bennonis	X	DSM 23058	BEN	BEN_DSM 23058	Draft (87)	AQWR00000000
bennonis		JCM 16335	BEN	BEN_JCM 16335	Draft (242)	BAME00000000
canoris		COT-108 OH1224	CAN	CAN_COT-108 OH1224	Draft (21)	JQZX00000000
canoris	X	COT-108 OH2762	CAN	CAN_OH2762	Draft (14)	JQZV00000000
sp.		COT-108_OH1349	CAN	CAN_COT-108_OH1349	Draft (43)	JRAH00000000
sp.		COT-108_OH2963	CAN	CAN_COT-108_OH2963	Draft (21)	JRAP00000000
catoniae		ATCC 51270	CAT	CAT_ATCC 51270	Draft (25)	JDFF00000000
catoniae	X	F0037	CAT	CAT_F0037	Draft (18)	AMEQ00000000
cangingivalis		ATCC 700135	CGI	CGI_ATCC 700135	Draft (34)	FUWL00000000
cangingivalis		COT-109 OH1379	CGI	CGI_OH1379	Draft (21)	JQJF00000000
cangingivalis		COT-109 OH1386	CGI	CGI_OH1386	Draft (65)	JQJD00000000
cangingivalis		JCM 15983	CGI	CGI_JCM 15983	Draft (48)	BAKR00000000
cangingivalis	X	NCTC 12856	CGI	CGI_NCTC12856	Complete (1)	LR134506
cangingivalis		NCTC 12857	CGI	CGI_NCTC12857	Draft (14)	UATO01000000
circumdentaria	X	DSM 13022	CIR	CIR_DSM_103022	Draft (30)	JACIJQ010000001-07
circumdentaria		ATCC 51356	CIR	CIR_ATCC 51356	Draft (35)	FUXE00000000
crevioricanis		ATCC 55563	CRE	CRE_ATCC 55563	Draft (29)	FUXH00000000
crevioricanis		COT-253_OH1447	CRE	CRE_OH1447	Draft (30)	JQJC00000000
crevioricanis		COT-253_OH2125	CRE	CRE_OH2125	Draft (14)	JQJB00000000
crevioricani		JCM 13913	CRE	CRE_JCM 13913	Draft (89)	BAOV00000000
crevioricanis		JCM 15906	CRE	CRE_JCM 15906	Draft (118)	BAOU00000000
crevioricanis	X	NCTC12858	CRE	CRE_NCTC12858	Complete (1)	LS483447
endodontalis		ATCC 35406	END	END_ATCC 35406	Draft (37)	ACNN00000000
endodontalis		NCTC13058	END	END_NCTC13058	Draft (2)	UGTE01000000
endodontalis	X	FDAARGOS_1506	END	END_FDAARGOS_1506	Complete (1)	ASM2009737
gingivicanis	X	COT-022 OH1391	GGI	GGI_JCM 15907	Draft (19)	JQZW00000000

List of *Porphyromonas* (and related information) used in this study

Species	Ref	Strain	Group acronym (this study)	Acronym (this study)	Genome Status (nb contigs)	Accession (WGS record)
gingivicanis		JCM 1597	GGI	GGI_OH1391	Draft (35)	BAKX00000000
gingivalis		3_3	GIN	GIN_3_3	Draft (72)	FUFB00000000
gingivalis		3A1	GIN	GIN_3A1	Draft (56)	FUFC00000000
gingivalis		7BTORR	GIN	GIN_7BTORR	Draft (72)	FUFD00000000
gingivalis		11A	GIN	GIN_11A	Draft (89)	FUFE00000000
gingivalis		13_1	GIN	GIN_13-1	Draft (68)	FUGG00000000
gingivalis		15_9	GIN	GIN_15_9	Draft (68)	FUGF00000000
gingivalis		84_3	GIN	GIN_84-3	Draft (50)	FUFG00000000
gingivalis		381	GIN	GIN_381	Complete (1)	CP012889
gingivalis		381OKJP	GIN	GIN_381OKJP	Draft (127)	QPGS01000000
gingivalis		A7A1-28	GIN	GIN_A7A1-28	Complete (1)	CP013131
gingivalis		A7436	GIN	GIN_A7436	Complete (1)	CP011995
gingivalis		AFR5B1	GIN	GIN_AFR5B1	Draft (88)	FUFJ00000000
gingivalis		AJW4	GIN	GIN_AJW4	Complete (1)	CP011996
gingivalis		Ando	GIN	GIN_Ando	Draft (112)	BCBV01000000
gingivalis	X	ATCC 33277	GIN	GIN_ATCC 33277	Complete (1)	CP025930/AP009380
gingivalis		ATCC 49417	GIN	GIN_ATCC 49417	Draft (77)	FUFH00000000
gingivalis		CP3	GIN	GIN_CP3	Draft (118)	SGBA01000000
gingivalis		F0185	GIN	GIN_F0185	Draft (113)	AWVC00000000
gingivalis		F0566	GIN	GIN_F0566	Draft (192)	AWVD00000000
gingivalis		F0568	GIN	GIN_F0568	Draft (154)	AWUU00000000
gingivalis		F0569	GIN	GIN_F0569	Draft (111)	AWUV00000000
gingivalis		F0570	GIN	GIN_F0570	Draft (117)	AWUW00000000
gingivalis		H3	GIN	GIN_H3	Draft (165)	SGAZ01000000
gingivalis		HG66	GIN	GIN_HG66	Complete (1)	CP007756.1
gingivalis		JCVI SC001	GIN	GIN_JCVI SC001	Draft (282 gaps)	APMB01000000
gingivalis		KCOM 2796	GIN	GIN_KCOM 2796	Complete (1)	CP024597
gingivalis		KCOM 2797	GIN	GIN_KCOM 2797	Draft (44)	NHRU00000000
gingivalis		KCOM 2798	GIN	GIN_KCOM 2798	Complete (1)	CP024598
gingivalis		KCOM 2799	GIN	GIN_KCOM 2799	Complete (1)	CP024601
gingivalis		KCOM 2800	GIN	GIN_KCOM 2800	Complete (1)	CP024599

List of *Porphyromonas* (and related information) used in this study

Species	Ref	Strain	Group acronym (this study)	Acronym (this study)	Genome Status (nb contigs)	Accession (WGS record)
gingivalis		KCOM 2801	GIN	GIN_KCOM 2801	Complete (1)	CP024600
gingivalis		KCOM 2802	GIN	GIN_KCOM 2802	Complete (1)	CP024591
gingivalis		KCOM 2803	GIN	GIN_KCOM 2803	Complete (1)	CP024592
gingivalis		KCOM 2804	GIN	GIN_KCOM 2804	Complete (1)	CP024593
gingivalis		KCOM 2805	GIN	GIN_KCOM 2805	Complete (1)	CP024594
gingivalis		KCOM 3001	GIN	GIN_KCOM 3001	Complete (1)	CP024595
gingivalis		KCOM 3131	GIN	GIN_KCOM 3131	Complete (1)	CP024596
gingivalis		MP4-504	GIN	GIN_MP4-504	Draft (92)	LOEL00000000
gingivalis		SJD2	GIN	GIN_SJD2	Draft (117)	ASYL00000000
gingivalis		SJD4	GIN	GIN_SJD4	Draft (147)	KZ248242-KZ248388
gingivalis		SJD5	GIN	GIN_SJD5	Draft (194)	ASYN00000000
gingivalis		SJD11	GIN	GIN_SJD11	Draft (156)	KZ248389-KZ248544
gingivalis		SJD12	GIN	GIN_SJD12	Draft (146)	KZ253969-KZ254114
gingivalis		SU60	GIN	GIN_SU60	Draft (53)	FUFI00000000
gingivalis		TDC60	GIN	GIN_TDC60	Complete (1)	CP025931/AP012203
gingivalis		W50	GIN	GIN_W50	Draft (104)	AJZS00000000
gingivalis		W83	GIN	GIN_W83	Complete (1)	CP025932/AE015924
gingivalis		W4087	GIN	GIN_W4087	Draft (114)	AWVE00000000
gingivalis		WW2096	GIN	GIN_WW2096	Draft (116)	NSLX01000000
gingivalis		WW2842	GIN	GIN_WW2842	Draft (95)	NSLW01000000
gingivalis		WW2866	GIN	GIN_WW2866	Draft (123)	NSLV01000000
gingivalis		WW2881	GIN	GIN_WW2881	Draft (484)	NSLU01000000
gingivalis		WW2885	GIN	GIN_WW2885	Draft (196)	NSLT01000000
gingivalis		WW2903	GIN	GIN_WW2903	Draft (115)	NSLS01000000
gingivalis		WW2931	GIN	GIN_WW2931	Draft (103)	NSLR01000000
gingivalis		WW2952	GIN	GIN_WW2952	Draft (162)	NSLQ01000000
gingivalis		WW3039	GIN	GIN_WW3039	Draft (132)	NSLN01000000
gingivalis		WW3102	GIN	GIN_WW3102	Draft (149)	NSLO01000000
gingivalis		WW5127	GIN	GIN_WW5127	Draft (119)	NSLL01000000
gulae		COT-052_OH1355	GUL	GUL_OH1355	Draft (40)	JRAG00000000
gulae		COT-052_OH1451	GUL	GUL_OH1451	Draft (89)	JRAI00000000

List of *Porphyromonas* (and related information) used in this study

Species	Ref	Strain	Group acronym (this study)	Acronym (this study)	Genome Status (nb contigs)	Accession (WGS record)
gulae		COT-052_OH2179	GUL	GUL_OH2179	Draft (26)	JRAJ00000000
gulae		COT-052_OH2199	GUL	GUL_OH2199	Draft (92)	JRAE01000000
gulae		COT-052_OH2857	GUL	GUL_OH2857	Draft (53)	JRFD00000000
gulae		COT-052_OH3439	GUL	GUL_OH3439	Draft (163)	JRAK00000000
gulae		COT-052_OH3471	GUL	GUL_OH3471	Draft (44)	JRAQ00000000
gulae		COT-052_OH3498	GUL	GUL_OH3498	Draft (71)	JRAF00000000
gulae	X	COT-052_OH3856	GUL	GUL_OH3856	Draft (31)	JRAT00000000
gulae		COT-052_OH4119	GUL	GUL_OH4119	Draft (52)	JRAL00000000
gulae		DSM 15663	GUL	GUL_DSM 15663	Draft (63)	ARJN00000000
gulae		OH3161B	GUL	GUL_OH3161B	Draft (47)	JQJE00000000
sp.		UQD_349_COT-052_OH4946	GUL	GUL_OH4946	Draft (34)	JQZY00000000
levii		AF0918	LEV	LEV_AF0918	Draft (364)	SPNB01000000
levii		AF5678	LEV	LEV_AF5678	Draft (468)	SPNC01000000
levii	X	DSM 23370	LEV	LEV_DSM 23370	Draft (124)	ARBX00000000
loveana	X	DSM 28520	LOV	LOV_DSM 28520	Draft (39)	QEKY01000000
macacae		COT-192_OH2631	MAC	MAC_OH2631	Draft (48)	JRFB00000000
macacae		COT-192_OH2859	MAC	MAC_OH2859	Draft (32)	JRFA00000000
macacae	X	DSM 20710	MAC	MAC_DSM 20710	Draft (31)	ARBY00000000
macacae		JCM 13914	MAC	MAC_JCM 13914	Draft (44)	BAKQ00000000
macacae		JCM 15984	MAC	MAC_JCM 15984	Draft (69)	BAKS00000000
macacae		NCTC11632	MAC	MAC_NCTC11632	Draft (6)	UGTF01000000
macacae		NCTC13100	MAC	MAC_NCTC13100	Draft (5)	UGTI01000000
pasteri	X	JCM 30531	PAS	PAS_JCM 30531	Draft (9)	ASM1464775
somerae		DSM 23387 = JCM 13868	SOM	SOM_DSM_23387	Draft (95)	AQVC00000000
somerae	X	CE91-St14	SOM	SOM_St14	Complete (1)	AP025559
sp.		UMGS1452	UEN	UEN_UMGS1452	Draft (337)	URXQ01000000
uenonis	X	DSM23387	UEN	UEN_DSM23387	Draft (44)	AXVC00000000
uenonis		JCM13868	UEN	UEN_JCM13868	Draft (112)	BAJM00000000
sp.		COT-290_OH3588	no assignation	PSP_OH3588	Draft (48)	JRFC00000000

List of *Porphyromonas* (and related information) used in this study

Species	Ref	Strain	Group acronym (this study)	Acronym (this study)	Genome Status (nb contigs)	Accession (WGS record)
sp.		UMGS18	no assignation	PSP_UMGS18	Draft (60)	UWSO01000000
sp.		UMGS107	no assignation	PSP_UMGS107	Draft (65)	UQBE01000000
sp.		UMGS166	no assignation	PSP_UMGS166	Draft (83)	UQDF01000000
sp.		UMGS907	no assignation	PSP_UMGS907	Draft (109)	URDQ01000000
sp.		UMGS1085	no assignation	PSP_UMGS1085	Draft (286)	URKO01000000
sp.		60-3	**not *uenonis***	PSP_60-3	Draft (250)	ACLR00000000
sp.		31_2	***Parabacteroides***	not considered	Draft (13)	ACUD00000000
sp.		bin_26	***Genomes mixture***	not considered	Draft (328	RBKG01000000
somerae		KA00683	***Genomes mixture***	not considered	Draft (62)	LSDK00000000
sp.		CAG:1061	***Genomes mixture***	not considered	Draft (344)	CAXO000000000
sp.		oral taxon 279 str. F0450	***Genomes mixture***	not considered	Draft (51)	ALKJ00000000
sp.		HMSC065F10	***Genomes mixture***	not considered	Draft (103)	LTXH00000000
sp.		HMSC077F02	***Genomes mixture***	not considered	Draft (85)	LTSX00000000
sp.		KLE 1280	***Genomes mixture***	not considered	Draft (8)	JNOS00000000
sp.		UMGS2020	***Genomes mixture***	not considered	Draft (280)	USRR01000000
sp.		COT-290_OH860	***Genomes mixture***	not considered	Draft (82)	JRAR00000000
sp.		COT-239_OH1446	***Genomes mixture***	not considered	Draft (37)	JRAO00000000
sp.		UMGS547	***Genomes mixture***	not considered	Draft (46)	UQQP01000000
sp.		UMGS338	***Genomes mixture***	not considered	Draft (33)	UQJH01000000
sp.		UMGS713	***Genomes mixture***	not considered	Draft (163)	UQWO01000000
sp.		UMGS1769	***Genomes mixture***	not considered	Draft (183)	USJF01000000
sp.		UMGS2040	***Genomes mixture***	not considered	Draft (238)	USSQ01000000
sp.		oral taxon 278 str. W7784	***Genomes mixture***	not considered	Draft (38)	AWUX00000000
sp.		MGYG-HGUT-04270	***Genomes mixture***	not considered	Draft (197)	CABPCS010000001-07

Table S1. List of *Porphyromonas* genomes used in this study. Genomes were grouped into clades following genomic data-driven taxonomic clustering. Complete genomes are in green, *Porphyromonas* sp. grouped in a clade are in blue, a mislabelled *P. somerae* is indicated in yellow, *P.* sp. genomes that could not be grouped with others are in dark grey and mislabelled "*Porphyromonas*" genomes are in purple. All accession numbers are indicated as well as the strain.

Acronym (this study)	HMM groups	Locus_tag	Old_locus_tag	Initiation codon reannotation	SignalP-6/LipoP prediction before curation	SignalP-6/LipoP prediction after curation	Size before biocuration (in aa)	Size after biocuration (in aa)	Lipoprotein signal peptide (Sec/SPII)	Cleavage site between pos.	Size after clivage (in aa)
ASA_PR426713P-I	Ffp1_A	HMPREF9294_RS07130	HMPREF9294_0374	VTLRL… (position +38aa, 5' truncation)	Cytoplasmic	SpII	543	506	VTLRLISLVVCMSVLLLGS	19 and 20: LGS-CR	487
ASA_DSM 20707	Ffp1_A	PORAS_RS06500	Poras_1299	VTPRL… (position +49aa, 5' truncation)	Cytoplasmic	SpII	553	505	VTPRLISLVVCMSVLLLGS	19 and 20: LGS-CR	486
BEN_DSM 23058	Ffp1_B	B088_RS06165	-	-	SpII	-	525	-	MKRLKSTLLASIALLAVG	18 and 19:AVG-CS	507
BEN_JCM 16335	Ffp1_B	fig|1236537.5.peg.6498	-	-	SpII	-	517	-	MKRLKSTLLASIALLAVG	18 and 19:AVG-CS	499
CAN_COT-108 OH1224	Ffp1_B	HQ29_RS00605	HQ29_00650	-	SpII	-	527	-	MRKSHISALVFALALFSSG	19 and 20:SSG-CK	508
CAN_OH2762	Ffp1_B	HQ43_RS08510	HQ43_09055	-	SpII	-	527	-	MRKSHISALVFALALFSSG	19 and 20:SSG-CK	508
CAN_OH1349	Ffp1_B	JT26_RS08750	JT26_09370	-	SpII	-	527	-	MRKSHISALVLALALFSSG	19 and 20:SSG-CK	508
CAN_OH2963	Ffp1_B	HQ39_00925	-	-	SpII	-	527	-	MRKSHISALVFALALFSSG	19 and 20:SSG-CK	508
CAT_ATCC 51270	Ffp1_A	HMPREF0636_1008	-	MKPS….(position -8 aa, 5' elongation)	Cytoplasmic	SpII	467	475	MKPSLSLIMVLALAFSS	17 and 18: FSS-CT	458
CAT_F0037	Ffp1_A	HMPREF9134_01065	-	MKPS….(position -8 aa, 5' elongation)	Cytoplasmic	SpII	467	475	MKPSLSLIMVLALAFSS	17 and 18: FSS-CT	458
CGI_ATCC 700135	Ffp1_B	B5D10_RS02040	SAMN02745205_00414	-	SpII	-	501	-	MKRPIKGLAILILLCVLVA	19 and 20:LVA-CN	482
CGI_OH1379	Ffp1_B	HQ34_RS02105	HQ34_02210	-	SpII	-	501	-	MKRPIKGLTILVLLCVLVA	19 and 20:LVA-CN	482
CGI_OH1386	Ffp1_B	HQ35_RS07210	HQ35_07500	-	SpII	-	501	-	MKRPIKGLTILVLLCVLVA	19 and 20:LVA-CN	482
CGI_JCM 15983	Ffp1_B	JCM15983_RS07445	-	-	SpII	-	501	-	MKRPIKGLAILILLCVLVA	19 and 20:LVA-CN	482
CGI_NCTC12856	Ffp1_B	EL262_RS03910	NCTC12856_00820	-	SpII	-	501	-	MKRPIKGLAILILLCVLVA	19 and 20:LVA-CN	482
CGI_NCTC12857	Ffp1_B	NCTC12857_00769	-	-	SpII	-	501	-	MKRPIKGLAILILLCVLVA	19 and 20:LVA-CN	482
CIR_DSM_103022	Ffp1_A	Ga0373368_1702	-	LFFH …(position -55 aa, 5' elongation)	Cytoplasmic	SpII (best score)	439	493	LFFHIPLLSLFILSLLS	17 and 18:LLS-CT	476
CIR_ATCC 51356	Ffp1_A	SAMN02745171_01593	-	LFFH …(position +4 aa, 5' truncation)	SpII	SpII (best score)	497	493	LFFHIPLLSLFILSLLS	17 and 18:LLS-CT	476
CRE_ATCC 55563	Ffp1_A	B5D48_RS07615	SAMN02745203_01515	LFRI …(position +4 aa, 5' truncation)	SpII	SpII (best score)	485	-	LFRIWLLPVVFVLAMG	16 and 17:AMG-CS	465
CRE_OH1447	Ffp1_A	HQ38_00070	-	LFRI …(position +4 aa, 5' truncation)	SpII	SpII (best score)	485	-	LFRIWLLPVVFVLAMG	16 and 17:AMG-CS	465
CRE_OH2125	Ffp1_A	HQ45_07910	-	LFRI …(position +4 aa, 5' truncation)	SpII	SpII (best score)	485	-	LFRIWLLPVVFVLAMG	16 and 17:AMG-CS	465
CRE_JCM 13913	Ffp1_A	PORCAN_38	-	LFRI …(position +4 aa, 5' truncation)	SpII	SpII (best score)	485	-	LFRIWLLPVVFVLAMG	16 and 17:AMG-CS	465
CRE_JCM 15906	Ffp1_A	TX94_RS03855	PORCRE_924	LFRI …(position +4 aa, 5' truncation)	SpII	SpII (best score)	485	-	LFRIWLLPVVFVLAMG	16 and 17:AMG-CS	465
CRE_NCTC12858	Ffp1_A	DQN70_RS01465	NCTC12858_00290	LFRI …(position +4 aa, 5' truncation)	SpII	SpII (best score)	485	-	LFRIWLLPVVFVLAMG	16 and 17:AMG-CS	465
END_ATCC 35406	Ffp1_A	POREN0001_RS08405	POREN0001_0653	-	SpII	-	490	-	MKSTAKILFGGLFMGVLLLA	20 and 21:LLA-CH	470
END_NCTC13058	Ffp1_A	NCTC13058_00879	-	-	SpII	-	490	-	MKSTAKILFGGLFMGVLLLA	20 and 21:LLA-CH	470
END_FDAARGOS_1506	Ffp1_A	LA319_RS03360	LA319_03360	-	SpII	-	490	-	MKSTAKILFGGLFMGVLLLA	20 and 21:LLA-CH	470
GGI_JCM 15907	Ffp1_A	JCM15907_RS02095	-	LAYR…(position +4 aa, 5' truncation)	SpII	-	495	491	LAYRFISILLFISFLGS	17 and 18:LGS-CK	474
GGI_OH1391	Ffp1_A	HQ36_RS03135	HQ36_03345	LAYR…(position +4 aa, 5' truncation)	SpII	-	495	491	LAYRFISILLFISFLGS	17 and 18:LGS-CK	474
GIN_3_3	Ffp1_A	PGIN_3-3_01611	-	-	SpII	-	481	-	MLTKLKTLLLGCSLACIGFS	20 and 21: GFS-CS	461
GIN_3A1	Ffp1_A	B0496_RS06505	PGIN_3A1_01536	-	SpII	-	481	-	MLTKLKTLLLGCSLACIGFS	20 and 21: GFS-CS	461
GIN_7BTORR	Ffp1_A	PGIN_7BTORR_01660	-	-	SpII	-	481	-	MLTKLKTLLLGCSLACIGFS	20 and 21: GFS-CS	461
GIN_11A	Ffp1_A	PGIN_11A_01261	-	-	SpII	-	481	-	MLTKLKTLLLGCSLACIGFS	20 and 21: GFS-CS	461
GIN_13-1	Ffp1_A	PGIN_13-1_00894	-	-	SpII	-	481	-	MLTKLKTLLLGCSLACIGFS	20 and 21: GFS-CS	461
GIN_15_9	Ffp1_A	CEP61_RS05480	PGIN_15-9_01143	-	SpII	-	481	-	MLTKLKTLLLGCSLACIGFS	20 and 21: GFS-CS	461
GIN_84-3	Ffp1_A	PGIN_84-3_01869	-	-	SpII	-	481	-	MLTKLKTLLLGCSLACIGFS	20 and 21: GFS-CS	461
GIN_381	Ffp1_A	PGF_RS08670	PGF_00017830	-	SpII	-	481	-	MLTKLKTLLLGCSLACIGFS	20 and 21: GFS-CS	461
GIN_381OKJP	Ffp1_A	DOE52_RS07275	DOE52_07370	-	SpII	-	481	-	MLTKLKTLLLGCSLACIGFS	20 and 21: GFS-CS	461
GIN_A7A1-28	Ffp1_A	PGS_00016750	-	-	SpII	-	481	-	MLTKLKTLLLGCSLACIGFS	20 and 21: GFS-CS	461
GIN_A7436	Ffp1_A	PGA7_RS08340	PGA7_00017440	-	SpII	-	481	-	MLTKLKTLLLGCSLACIGFS	20 and 21: GFS-CS	461
GIN_AFR5B1	Ffp1_A	PGIN_AFR-5B1_00930	-	-	SpII	-	481	-	MLTKLKTLLLGCSLACIGFS	20 and 21: GFS-CS	461
GIN_AJW4	Ffp1_A	PGJ_RS08140	PGJ_00016930	-	SpII	-	481	-	MLTKLKTLLLGCSLACIGFS	20 and 21: GFS-CS	461
GIN_Ando	Ffp1_A	PGANDO_1295	-	-	SpII	-	481	-	MLTKLKTLLLGCSLACIGFS	20 and 21: GFS-CS	461
GIN_ATCC 33277	Ffp1_A	PGN_RS08575	PGN_1808	-	SpII	-	481	-	MLTKLKTLLLGCSLACIGFS	20 and 21: GFS-CS	461
GIN_ATCC 49417	Ffp1_A	PGIN_ATCC49417_00365	-	-	SpII	-	481	-	MLTKLKTLLLGCSLACIGFS	20 and 21: GFS-CS	461
GIN_CP3	Ffp1_A	EW639_RS04185	EW639_04180	-	SpII	-	481	-	MLTKLKTLLLGCSLACIGFS	20 and 21: GFS-CS	461
GIN_F0185	Ffp1_A	HMPREF1988_00474	-	-	SpII	-	481	-	MLTKLKTLLLGCSLACIGFS	20 and 21: GFS-CS	461
GIN_F0566	Ffp1_A	HMPREF1989_01686	-	-	SpII	-	481	-	MLTKLKTLLLGCSLACIGFS	20 and 21: GFS-CS	461
GIN_F0568	Ffp1_A	HMPREF1553_02200	-	-	SpII	-	481	-	MLTKLKTLLLGCSLACIGFS	20 and 21: GFS-CS	461
GIN_F0569	Ffp1_A	HMPREF1554_01931	-	-	SpII	-	481	-	MLTKLKTLLLGCSLACIGFS	20 and 21: GFS-CS	461
GIN_F0570	Ffp1_A	HMPREF1555_00703	-	-	SpII	-	481	-	MLTKLKTLLLGCSLACIGFS	20 and 21: GFS-CS	461
GIN_H3	Ffp1_A	EW638_RS01795	EW638_01795	-	SpII	-	481	-	MLTKLKTLLLGCSLACIGFS	20 and 21: GFS-CS	461
GIN_HG66	Ffp1_A	EG14_RS00370	EG14_00390	-	SpII	-	481	-	MLTKLKTLLLGCSLACIGFS	20 and 21: GFS-CS	461
GIN_JCVI SC001	Ffp1_A	A343_0931	-	-	SpII	-	481	-	MLTKLKTLLLGCSLACIGFS	20 and 21: GFS-CS	461
GIN_KCOM 2796	Ffp1_A	CS059_RS02825	CS059_02815	-	SpII	-	481	-	MLTKLKTLLLGCSLACIGFS	20 and 21: GFS-CS	461
GIN_KCOM 2797	Ffp1_A	CBG53_RS04765	CBG53_04755	-	SpII	-	481	-	MLTKLKTLLLGCSLACIGFS	20 and 21: GFS-CS	461
GIN_KCOM 2798	Ffp1_A	CS374_RS06220	CS374_06175	-	SpII	-	481	-	MLTKLKTLLLGCSLACIGFS	20 and 21: GFS-CS	461
GIN_KCOM 2799	Ffp1_A	CS387_RS05960	CS387_05925	-	SpII	-	481	-	MLTKLKTLLLGCSLACIGFS	20 and 21: GFS-CS	461
GIN_KCOM 2800	Ffp1_A	CS388_RS06295	CS388_06250	-	SpII	-	481	-	MLTKLKTLLLGCSLACIGFS	20 and 21: GFS-CS	461
GIN_KCOM 2801	Ffp1_A	CS543_RS02035	CS543_02040	-	SpII	-	481	-	MLTKLKTLLLGCSLACIGFS	20 and 21: GFS-CS	461
GIN_KCOM 2802	Ffp1_A	CS544_RS06860	CS544_06820	-	SpII	-	481	-	MLTKLKTLLLGCSLACIGFS	20 and 21: GFS-CS	461
GIN_KCOM 2803	Ffp1_A	CS545_RS01100	CS545_01090	-	SpII	-	481	-	MLTKLKTLLLGCSLACIGFS	20 and 21: GFS-CS	461
GIN_KCOM 2804	Ffp1_A	CS546_RS04285	CS546_04255	-	SpII	-	481	-	MLTKLKTLLLGCSLACIGFS	20 and 21: GFS-CS	461
GIN_KCOM 2805	Ffp1_A	CS548_RS00115	CS548_00110	-	SpII	-	481	-	MLTKLKTLLLGCSLACIGFS	20 and 21: GFS-CS	461
GIN_KCOM 3001	Ffp1_A	CS550_RS00280	CS550_00280	-	SpII	-	481	-	MLTKLKTLLLGCSLACIGFS	20 and 21: GFS-CS	461
GIN_KCOM 3131	Ffp1_A	CS549_RS03615	CS549_03580	-	SpII	-	481	-	MLTKLKTLLLGCSLACIGFS	20 and 21: GFS-CS	461
GIN_MP4-504	Ffp1_A	AT291_01865	-	-	SpII	-	481	-	MLTKLKTLLLGCSLACIGFS	20 and 21: GFS-CS	461
GIN_SJD2	Ffp1_A	SJDPG2_RS03990	SJDPG2_06325	-	SpII	-	481	-	MLTKLKTLLLGCSLACIGFS	20 and 21: GFS-CS	461
GIN_SJD4	Ffp1_A	SJDPG4_RS07350	SJDPG4_07395	-	SpII	-	481	-	MLTKLKTLLLGCSLACIGFS	20 and 21: GFS-CS	461
GIN_SJD5	Ffp1_A	SJDPG5_RS08005	SJDPG5_07225	-	SpII	-	481	-	MLTKLKTLLLGCSLACIGFS	20 and 21: GFS-CS	461
GIN_SJD11	Ffp1_A	SJDPG11_RS03850	SJDPG11_03950	-	SpII	-	481	-	MLTKLKTLLLGCSLACIGFS	20 and 21: GFS-CS	461
GIN_SJD12	Ffp1_A	SJDPG12_RS04055	SJDPG12_04100	-	SpII	-	481	-	MLTKLKTLLLGCSLACIGFS	20 and 21: GFS-CS	461
GIN_SU60	Ffp1_A	PGIN_YH522_00088	-	-	SpII	-	481	-	MLTKLKTLLLGCSLACIGFS	20 and 21: GFS-CS	461
GIN_TDC60	Ffp1_A	PGTDC60_RS00615	PGTDC60_0141	-	SpII	-	481	-	MLTKLKTLLLGCSLACIGFS	20 and 21: GFS-CS	461
GIN_W50	Ffp1_A	HMPREF1322_RS07675	HMPREF1322_0808	-	SpII	-	481	-	MLTKLKTLLLGCSLACIGFS	20 and 21: GFS-CS	461
GIN_W83	Ffp1_A	CF003_RS19550	CF003_1881	-	SpII	-	481	-	MLTKLKTLLLGCSLACIGFS	20 and 21: GFS-CS	461
GIN_W4087	Ffp1_A	HMPREF1990_01936	-	-	SpII	-	481	-	MLTKLKTLLLGCSLACIGFS	20 and 21: GFS-CS	461
GIN_WW2096	Ffp1_A	CLI72_04935	-	-	SpII	-	481	-	MLTKLKTLLLGCSLACIGFS	20 and 21: GFS-CS	461
GIN_WW2842	Ffp1_A	CLI73_05985	-	-	SpII	-	481	-	MLTKLKTLLLGCSLACIGFS	20 and 21: GFS-CS	461
GIN_WW2866	Ffp1_A	CLI74_08035	-	-	SpII	-	481	-	MLTKLKTLLLGCSLACIGFS	20 and 21: GFS-CS	461
GIN_WW2881	Ffp1_A	CLI75_RS00850	CLI75_00850	-	SpII	-	481	-	MLTKLKTLLLGCSLACIGFS	20 and 21: GFS-CS	461
GIN_WW2885	Ffp1_A	CLI76_07255	-	-	SpII	-	481	-	MLTKLKTLLLGCSLACIGFS	20 and 21: GFS-CS	461
GIN_WW2903	Ffp1_A	CLI77_RS05155	CLI77_05155	-	SpII	-	481	-	MLTKLKTLLLGCSLACIGFS	20 and 21: GFS-CS	461
GIN_WW2931	Ffp1_A	CLI78_RS08380	CLI78_08375	-	SpII	-	481	-	MLTKLKTLLLGCSLACIGFS	20 and 21: GFS-CS	461
GIN_WW2952	Ffp1_A	CLI79_07275	-	-	SpII	-	481	-	MLTKLKTLLLGCSLACIGFS	20 and 21: GFS-CS	461
GIN_WW3039	Ffp1_A	CLI82_RS08915	CLI82_08920	-	SpII	-	481	-	MLTKLKTLLLGCSLACIGFS	20 and 21: GFS-CS	461
GIN_WW3102	Ffp1_A	CLI81_06020	-	-	SpII	-	481	-	MLTKLKTLLLGCSLACIGFS	20 and 21: GFS-CS	461
GIN_WW5127	Ffp1_A	CLI84_RS06820	CLI84_06820	-	SpII	-	481	-	MLTKLKTLLLGCSLACIGFS	20 and 21: GFS-CS	461
GUL_OH1355	Ffp1_A	HQ42_RS07930	HQ42_08260	-	SpII	-	481	-	MLTKLKSLLLGCSFACVGFS	20 and 21: GFS-CS	461
GUL_OH1451	Ffp1_A	HR08_RS10055	HR08_10540	-	SpII	-	481	-	MLMKLKSLLLGCSLACIGFS	20 and 21: GFS-CS	461
GUL_OH2179	Ffp1_A	HR09_01945	-	-	SpII	-	481	-	MLTKLKSLLLGCSFACVGFS	20 and 21: GFS-CS	461
GUL_OH2199	Ffp1_A	HR10_RS02290	HR10_02360	-	SpII	-	481	-	MLTKLKSLLLGCSFACVGFS	20 and 21: GFS-CS	461
GUL_OH2857	Ffp1_A	HQ46_RS03870	HQ46_04080	-	SpII	-	481	-	MLTKLKSLLLGCSLACIGFS	20 and 21: GFS-CS	461
GUL_OH3439	Ffp1_A	HR15_RS03480	HR15_03625	-	SpII	-	481	-	MLMKLKSLLLGCSLACIGFS	20 and 21: GFS-CS	461
GUL_OH3471	Ffp1_A	HQ40_RS09880	HQ40_10345	-	SpII	-	481	-	MLMKLKSLLLGCSLACIGFS	20 and 21: GFS-CS	461
GUL_OH3498	Ffp1_A	HR16_RS01285	HR16_01335	-	SpII	-	481	-	MLTKLKSLLLGCSLACIGFS	20 and 21: GFS-CS	461
GUL_OH3856	Ffp1_A	HQ49_05395	-	-	SpII	-	481	-	MLMKLKSLLLGCSLACIGFS	20 and 21: GFS-CS	461
GUL_OH4119	Ffp1_A	HR17_RS03350	HR17_03500	-	SpII	-	481	-	MLTKLKSLLLGCSLACIGFS	20 and 21: GFS-CS	461
GUL_DSM 15663	Ffp1_A	F452_RS0101570	-	-	SpII	-	481	-	MLTKLKSLLLGCSFACVGFS	20 and 21: GFS-CS	461
GUL_OH3161B	Ffp1_A	HR13_RS00620	HR13_00630	-	SpII	-	481	-	MLTKLKSLLLGCSFACVGFS	20 and 21: GFS-CS	461
GUL_OH4946	Ffp1_A	HQ50_02910	-	-	SpII	-	481	-	MLTKLKSLLLGCSLACIGFS	20 and 21: GFS-CS	461
LEV_AF0918	Ffp1_B	E4P48_RS01530	E4P48_01535	-	SpII	-	514	-	MKTHLLCAIALLFLLLG	17 and 18:LLG-CN	497
LEV_AF5678	Ffp1_B	E4P47_RS07065	E4P47_07070	-	SpII	-	514	-	MKTHLLCAIALLFLLLG	17 and 18:LLG-CN	497
LEV_DSM 23370	Ffp1_B	A3GG_RS0105690	-	-	SpII	-	514	-	MKTHLLCAIALLFLLLG	17 and 18:LLG-CN	497
LOV_DSM 28520	Ffp1_A	C7382_RS00405	C7382_10187	-	SpII	-	481	-	MKLKLLFASLALMCLSIA	18 and 19:SIA-CS	463
MAC_OH2631	Ffp1_A	HR11_RS03275	HR11_03565	-	SpII	-	479	-	MKKHLLFVFTVFLIAISS	18 and 19:ISS-CN	461
MAC_OH2859	Ffp1_A	HQ47_RS07445	HQ47_07890	-	SpII	-	479	-	MKKHLLFVFTVFLIAISS	18 and 19:ISS-CN	461
MAC_DSM 20710	Ffp1_A	fig|1122974.3.peg.681	-	-	SpII	-	479	-	MKKHLLFVFALFLIAISS	18 and 19:ISS-CN	461
MAC_JCM 13914	Ffp1_A	fig|1236519.3.peg.1431	-	-	SpII	-	479	-	MKKHLLFVFALFLIAISS	18 and 19:ISS-CN	461
MAC_JCM 15984	Ffp1_A	JCM15984DRAFT_02252	-	note : this ORF is 3' truncated (end of contig)	SpII	-	464	-	…AISS (contig extremity)	na	460
MAC_NCTC11632	Ffp1_A	NCTC11632_00113	-	-	SpII	-	479	-	MKKHLLFVFTVFLIAISS	18 and 19:ISS-CN	461
MAC_NCTC13100	Ffp1_A	DXZ22_RS03080	NCTC13100_00629	-	SpII	-	479	-	MKKHLLFVFTVFLIAISS	18 and 19:ISS-CN	461
PAS_JCM 30531	Ffp1_A	IEX77_RS07640	GCM10007088_14930	MRRA…(position +10aa, 5' truncation)	SpII	SpII (best score)	486	476	MRRALSLSLSLLLLAG	16 and 17:LACG-CS	460
SOM_DSM_23387	Ffp1_B	A3GK_RS10475	-	note : this ORF is 3' truncated (end of contig)	SpII	-	483	-	MRRLLSIILLALIVSS	16 and 17:VSS-CN	467…
SOM_St14	Ffp1_B	CE91St14_06390	-	MRRL…(-20aa, 5' elongation)	Cytoplasmic	SpII	484	505	MRRLLSIILLALIVSS	16 and 17:VSS-CN	489
PSP_60-3	Ffp1_A	PORUE0001_RS06855	PORUE0001_1094	LATSK…(position +23, 5' truncation)	SpII	SpII (best score)	528	506	LATSKLISLILCMSVLLLGA	20 and 21:LGA-CR	486
PSP_OH3588	Ffp1_A	HQ48_RS05340	HQ48_05560	LTKR…(position +7, 5' truncated)	SpII	-	481	474	LTKRMVLAGMSIVVLAFA	18 and 19:AFA-CK	456
PSP_UMGS18	Ffp1_A	fig|159274.3.peg.1457	-	LISL…(position +28, 5' truncation)	SpII	SpII (best score)	529	501	LISLVLCMSVLLLGA	15 and 16:LGA-CR	486
PSP_UMGS107	Ffp1_A	fig|159274.4.peg.1387	-	LIPEL…(position -14 aa, ORF 5' elongation)	Other	SpII	490	504	LIPELSFALCISILMLGA	18 and 19:LGA-CR	486
PSP_UMGS166	Ffp1_A	fig|159274.5.peg.636	-	LKHAF…(position +31, 5' truncation)	Other	SpII	540	509	LKHAFTATPKLLSLVLCMSVLLLGA	25 and 26:LGA-CR	484
PSP_UMGS907	Ffp1_A	fig|159274.9.peg.1443	-	LKLTC…(position +35, 5' truncation)	Other	SpII	545	511	LKLTCTATARLIPLVLCTSILLLGA	25 and 26:LGA-CR	486
UEN_UMGS1452	Ffp1_A	fig|159274.11.peg.121	-	MPRLI…(position 24)	SpII	SpII (best score)	493	503	MPRLISLVLCMSVLLLGA	18 and 19:LGA-CR	485
UEN_DSM23387	Ffp1_A	L215_RS09265	-	MPRLI…(position -10 aa, ORF 5' elongation)	Other	SpII	493	503	MPRLISLVLCMSVLLLGA	18 and 19:LGA-CR	485
UEN_JCM13868	Ffp1_A	JCM13868_RS05230	-	MPRLI…(position -10 aa, ORF 5' elongation)	Other	SpII	527	503	MPRLISLVLCMSVLLLGA	18 and 19:LGA-CR	485

Table S2. List and details of all *ffp1* genes identified during this study. Information includes HMM group membership, old and new locus_tags, items related to start codon reannotation (when needed), cell localization predictions, peptide signal, and before and after cleavage sizes.
